# Vicarious learned helplessness: a translationally relevant novel model of stress contagion elucidating sex-dependent prefrontal cortex pathology

**DOI:** 10.3389/fnbeh.2026.1788847

**Published:** 2026-03-05

**Authors:** Shashikant Patel, Roli Kushwaha, Debiprasad Sinha, Kalyani Soren, Arvind Kumar, Sumana Chakravarty

**Affiliations:** 1 Department of Applied Biology, CSIR-Indian Institute of Chemical Technology, Hyderabad, India; 2 Academy of Scientific and Innovative Research, Ghaziabad, Uttar Pradesh, India; 3 CSIR-Centre for Cellular and Molecular Biology, Hyderabad, India

**Keywords:** BDNF, corticosterone, depression, learned helplessness, prefrontal cortex, sex differences, stress contagion, vicarious trauma

## Abstract

**Introduction:**

Vicarious trauma, the psychological distress from witnessing others’ suffering, is an increasingly recognized precursor to depression and anxiety. However, the underlying neurobiological mechanisms remain poorly understood and appear to be sex-dependent. This study investigated the behavioral, physiological, and molecular consequences of purely psychological stress using a novel rodent model of vicarious learned helplessness (VLH).

**Methods:**

Male and female C57BL/6J mice were used to establish VLH paradigm. Observer mice witnessed conspecifics receiving inescapable foot shocks through a partitioned chamber allowing multisensory interaction. Following 7 days of conditioning, behavioral assays assessed anxiety and depressive symptoms. Prefrontal cortex tissue was analyzed using RT-qPCR and immunoblotting to identify molecular alterations.

**Results:**

Vicarious stress induced depression phenotype in both sexes, characterized by active avoidance deficits, anhedonia and anxiety, comparable to direct physical trauma. Physiological assessments revealed hypothalamic-pituitary-adrenal (HPA) axis hyperactivity with elevated plasma corticosterone in both sexes. While molecular analysis showed shared downregulation of metabotropic glutamate receptor 2 (mGluR2) and elevated Il6 mRNA in the prefrontal cortex, distinct sexual dimorphism emerged. Males displayed specific deficits in neurotrophic support (*Bdnf* and BDNF) and glucocorticoid signaling (*Nr3c1*), whereas females exhibited impairments in social bonding pathways (*Oxtr*) and postsynaptic scaffolding proteins (PSD-95 and SHANK3).

**Discussion:**

Vicarious trauma is sufficient to drive depression-like pathology through distinct molecular trajectories in males and females. These findings are suggestive of the critical necessity for sex-specific therapeutic strategies when treating trauma-related psychiatric disorders.

## 1 Introduction

Depression has emerged as a significant global mental health concern. The disorder is clinically characterized by an array of debilitating symptoms, including enduring sadness, anhedonia, disrupted sleep and appetite, fatigue and psychomotor retardation (Nestler et al., 2002; Deady et al., 2022; Patel et al., 2025a). However, alongside affective and somatic symptoms, depression has been associated with impairments in various cognitive domains (Marvel and Paradiso, 2004; Arnsten, 2015; Li K. et al., 2016). Patients frequently report impaired concentration, indecisiveness, and a deep sense of hopelessness. To dissect the complex pathophysiology of depression, researchers rely on animal models that recapitulate specific aspects of the disorder. One of the most foundational and validated paradigms is learned helplessness, first described by Seligman and Maier in the 1970s (Seligman and Maier, 1967). This model emerged from experiments in which animals exposed to uncontrollable and inescapable aversive stimuli, subsequently failed to learn to escape the stimuli even when an escape route was made available. This learned passivity models cognitive and motivational deficits seen in human depression.

The concept of learned helplessness extends beyond direct personal experience. Clinical relevance of vicarious trauma is evident in the high rates of depression and burnout among caregivers, first responders, and populations exposed to media depictions of violence. The concept of vicarious learned helplessness (VLH) has been discussed in psychological contexts (DeVellis et al., 1978; Chambers and Hammonds, 2014) however, only a few investigations explored its neurobiological basis owing to scarcity of animal models that can recapitulate vicarious transmission of stress. Researchers have focused extensively on learned helplessness induced by direct, uncontrollable stress, leaving the social transmission of this specific cognitive state unexplored (Landgraf et al., 2015; Huang et al., 2021). VLH concept argues that helplessness can be acquired by observing others in distress. This concept derives from Albert Bandura’s social learning theory, where behaviors are acquired through observation without direct reinforcement (Bandura, 1969).

The neurobiological underpinnings of depression have traditionally been linked to the dysregulation of the Hypothalamic-Pituitary-Adrenal (HPA) axis (Lamers et al., 2013; Nakatake et al., 2020). While the HPA axis mediates the physiological symptoms of stress, the cognitive symptoms specifically the perception of uncontrollability and the failure to execute coping strategies implicate higher-order cortical regions. The Prefrontal Cortex (PFC) is critical for executive functions, including the evaluation of threat salience, the regulation of limbic structures like the amygdala, and the execution of goal-directed behaviors (Arnsten, 2015; Colyn et al., 2019). Evidence suggests that chronic stress leads to molecular and structural atrophy in the PFC, characterized by the retraction of dendritic arbors and the loss of synaptic density. While this has been established in direct stress models, it remains unknown whether vicarious stress is sufficient to drive similar pathological changes in the PFC. The objectives of this study were to develop and validate a mouse model of VLH using a modified foot-shock paradigm that restricts physical contact while allowing full sensory observation. We characterized the resulting behavioral profile, including measures of helplessness, anxiety, behavioral despair, and social interaction in both sexes and assessed the associated molecular changes in PFC. Our results demonstrate the validation of a VLH paradigm that effectively induces a depressive-like phenotype through the social observation of conspecific distress.

## 2 Materials and methods

### 2.1 Animals

A total of sixty adult C57BL/6J mice aged 6–8 weeks were used in this study, comprising 30 male and 30 female mice. Mice were housed in a temperature and humidity-controlled vivarium on a 12-h light/dark cycle with *ad libitum* access to standard rodent chow and water. All experimental procedures were conducted at CSIR-CCMB in accordance with institutional guidelines and were approved by the Institutional Animal Care and Ethics Committee.

### 2.2 Vicarious learned helplessness paradigm

The VLH paradigm was conducted in a modified Ugo Basile fear conditioning apparatus (Supplementary Figure 1). The chamber was modified by inserting a transparent glass separator with small holes, which divided the electric shock grid floor into two equal halves. This setup allowed for continuous visual and olfactory communication between the two compartments. One side of the chamber was insulated with a rubber pad to prevent the delivery of foot shocks. The other side was the active “Shock” compartment.

**FIGURE 1:**
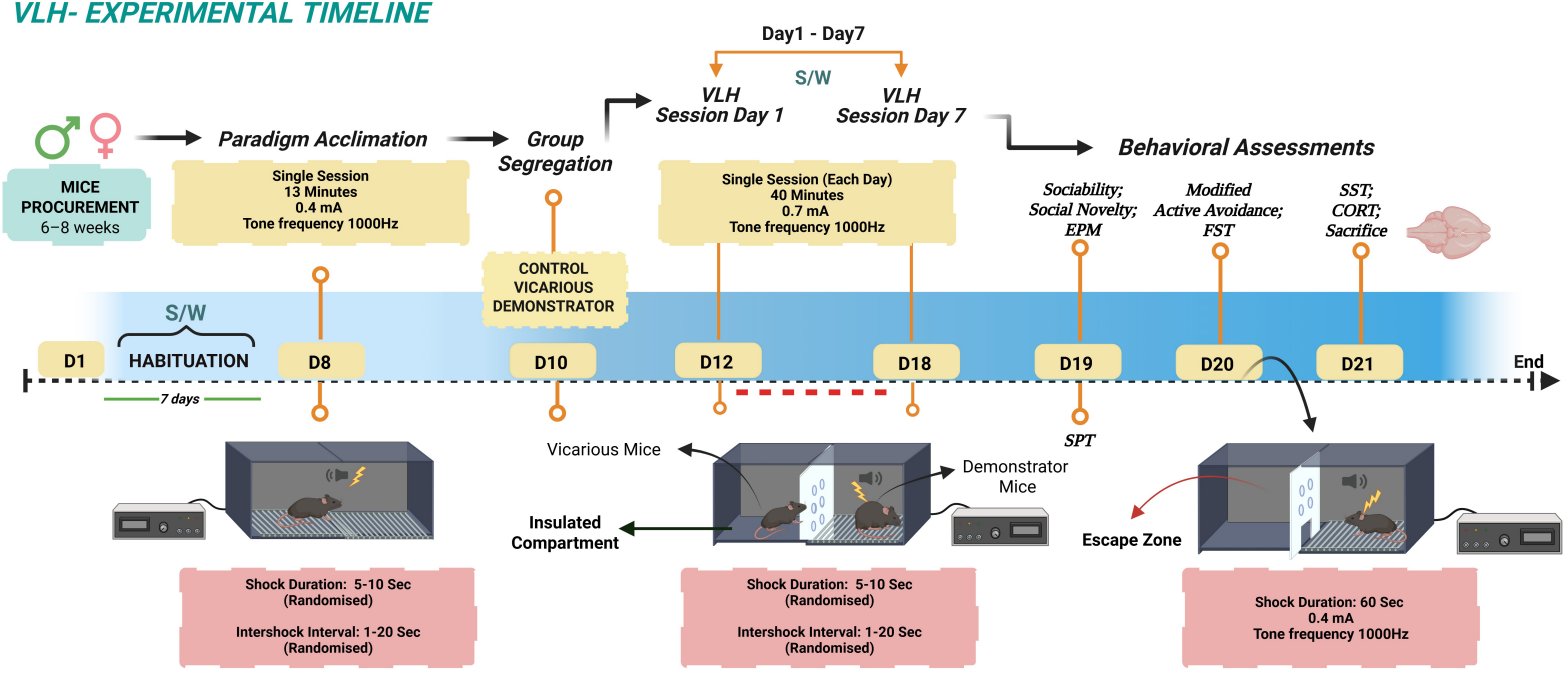
Experimental timeline and vicarious learned helplessness (VLH) protocol. The experimental timeline began with the procurement of male and female C57BL/6J mice aged 6-8 weeks followed by a habituation phase (days 1–7) where mice were acclimatized to sucrose and water access (S/W) with alternate bottle placement on consecutive days. On day 8, all animals underwent a single 13-minute paradigm acclimation session involving exposure to low-intensity foot shocks (0.4 mA) to habituate them to the apparatus context. Following group segregation on day 10 into control, vicarious, and demonstrator groups, the VLH paradigm was conducted over seven consecutive days (days 12–18). During these daily 40-min sessions, demonstrator mice received randomized inescapable foot shocks (0.7 mA, 5–10 s duration) paired with a 1,000 Hz auditory cue, while vicarious mice observed the stress from an adjacent insulated compartment. Behavioral assessments were performed on days 19, 20, and 21, including the Sucrose Preference Test, Elevated Plus Maze (EPM), Sociability and Social Novelty tests, Modified Active Avoidance, Forced Swim Test (FST), and Sucrose Splash Test (SST). The study concluded on day 21 with animal sacrifice and the collection of brain tissues for molecular analysis.

Following a 7-day habituation phase (Figure 1) to sucrose and water access (days 1–7), all mice underwent a single paradigm acclimation session on day 8 involving low-intensity foot shocks (0.4 mA, 13 min) to familiarize animals with the apparatus and contextual cues. Mice were then randomly assigned to one of three groups, control, vicarious and demonstrator. The VLH conditioning was conducted over seven consecutive days (days 12–18). During each daily 40-minute session, demonstrator mice received randomized inescapable foot shocks (0.7 mA, 5–10 s) paired with an auditory cue, while vicarious mice observed the procedure from an adjacent insulated compartment. Control mice underwent identical handling and cue exposure without shock delivery. After each session, the paired vicarious and demonstrator mice were housed overnight together in a standard cage that was divided by a transparent glass partition. Partitioned overnight housing was maintained to preserve continuous sensory exposure between demonstrator and observer mice while preventing physical interaction, a widely used approach in chronic social stress paradigms, to model sustained vicarious stress transmission. To ensure that the VLH phenotype reflected a generalized response to observed distress rather than familiarity with a single conspecific, vicarious mice were paired with a different demonstrator on each conditioning day. Control mice were housed under identical conditions in partitioned cages and were similarly rotated with different control partners across days. Behavioral assessments commenced 24 h after the final conditioning session and were conducted over three consecutive days (days 19–21), after which tissues from the same animals were collected for subsequent molecular analyses. Sample sizes for a few behavioral assays were < 10 either because all testing was required to be completed within a restricted 3-day window following the final stress session and prior to tissue collection. Exact n values are reported in the figure legends and results.

### 2.3 Behavioral assays

Twenty-four hours after VLH conditioning (day 19), mice underwent behavioral testing to assess anxiety and depression-like phenotypes. The battery began on day 19 with the assessment of social behavior (sociability and social novelty) followed by anxiety (elevated plus maze). On day 20, mice were subjected to modified active avoidance test and the forced swim test (FST), to evaluate vicarious learned helplessness and behavioral despair. On day 21 sucrose splash test (SST) was conducted for assessing deficits in self-care.

#### 2.3.1 Sucrose preference test

The SPT was used to assess anhedonia in the VLH paradigm. Prior to stress exposure, mice were habituated to a two-bottle choice setup, receiving alternating sucrose/water (S/W) and water/sucrose (W/S) positions daily to avoid side bias. During 7-day paradigm, mice were provided simultaneous access to 2% sucrose solution and water. Fluid intake was recorded on days 1, 3, 5, and 7 during the 7-day stress paradigm, and sucrose preference was calculated as:


$$\mathrm{Sucrose}\mathrm{Preference}\left(\%\right)=\frac{\mathrm{sucrose}\,\mathrm{intake}}{\left(\mathrm{sucrose}\,\mathrm{intake}+\mathrm{water}\,\mathrm{intake}\right)}\times 100$$

#### 2.3.2 Sociability and social novelty

The sociability and social novelty test were conducted as previously described (Lee et al., 2018) with minor modifications to assess social interaction deficits and social memory. The apparatus consisted of a rectangular open-field arena (40 × 60 cm) divided into two equal compartments by a central opaque partition, which left a passage open at one end to create a U-shaped access between sides. Identical cylindrical wire mesh cages were placed in the corner of each compartment to contain a stimulus mouse permitting visual, auditory, and olfactory contact. The test comprised three consecutive 5-min trials separated by brief inter-trial intervals during which the arena was cleaned with 70% ethanol. In the first trial (habituation), the test mouse freely explored the arena with empty enclosures to acclimate to the environment. During the second trial (sociability), an unfamiliar sex-matched conspecific (“Stranger 1”) was placed in one enclosure while the other remained empty; sociability was assessed by comparing the interaction time with the social stimulus versus the empty cage. For the third trial (social novelty), “Stranger 1” remained in its original position (now serving as the “Familiar” mouse), and a new unfamiliar mouse (“Novel”) was placed in the opposing cage. Interaction time was recorded to evaluate sociability in trial 2 and the preference for social novelty over the familiar conspecific in trial 3. Sociability and social novelty indices were calculated as percentages of interaction time.


Sociability(%)=[tStranger 1mice/(tStranger 1mice+tEmptycage)]×100

SocialNovelty(%)=[tNovelmice/(tNovelmice+tFamiliarmice)]×100

#### 2.3.3 Elevated plus maze

The EPM was used to assess anxiety-like behavior. The apparatus consisted of two open arms and two closed arms elevated above the floor. Each mouse was placed at the center facing an open arm and allowed to explore freely for 5 min. Behavior was recorded using a top mounted camera, and the time spent in open and closed arms, number of open-arm entries, and overall exploration patterns were quantified. Reduced open-arm exploration was interpreted as increased anxiety-like behavior.

#### 2.3.4 Modified active avoidance test

This test utilized the modified fear conditioning apparatus to directly measure the learned helplessness phenotype (Macheda et al., 2020). Before the first trial, each mouse was allowed to freely explore and move between the two compartments for 5 min with the shock turned off to ensure familiarization with the apparatus. Subsequently, each mouse underwent 20 recorded sessions. Mice were placed in the shock compartment and received a mild foot shock (0.4 mA) for a maximum duration of 60 s. Two seconds after the shock began, an automated slit door opened, allowing the mouse to escape into an insulated, shock-free compartment. The time taken by the mouse to reach the insulated compartment was scored as the escape latency. The total number of successful escapes (avoidances) was also recorded. For escape latency analyses, trials in which animals failed to reach the insulated compartment were treated as right-censored observations and assigned the predefined ceiling latency of 60 s, corresponding to the maximal response window of the task. This ensured uniform trial structure across animals, allowing latency measures to be analyzed within a bounded time framework consistent with avoidance-learning paradigms.

#### 2.3.5 Forced swim test

The FST was performed as per (Patel et al., 2026) to evaluate behavioral despair following stress exposure. Mice were individually placed in a transparent cylindrical tank (20 cm height, 15 cm diameter) filled with water maintained at 25 ± 1°C. Each session lasted 5 min and was video-recorded. Total immobility duration was scored. Immobility was defined as the absence of active swimming, with only minimal movements required to keep the head above water. Increased immobility was considered indicative of enhanced despair-like behavior.

#### 2.3.6 Sucrose splash test

The SST was performed as per (Patel et al., 2026) to assess motivational deficits in self-care behavior associated with depressive-like states. A 20% sucrose solution was gently sprayed onto the dorsal coat of each mouse to induce grooming. Immediately after spraying, mice were placed individually in clean cages, and behavior was recorded for 5 min. Latency to initiate grooming and total grooming duration were measured by an observer blinded to experimental conditions. Grooming was defined as any licking, pawing, or scratching directed toward the coat. Reduced grooming behavior was interpreted as decreased motivational drive.

### 2.4 Tissue collection and preparation

Following the conclusion of behavioral testing, mice were euthanized by cervical dislocation. For molecular analysis, brains were rapidly dissected, and specific brain areas were isolated as per Allen mouse brain atlas and immediately flash-frozen in liquid nitrogen, and stored at –80°C until further processing.

### 2.5 Serum corticosterone measurement

Serum corticosterone was quantified using a competitive ELISA kit (ADI-901-097, Enzo Life Sciences) according to the manufacturer’s protocol. Briefly, 10 μL serum was incubated with steroid displacement reagent (1:100) to release protein-bound hormone, diluted in assay buffer, and loaded onto plates pre-coated with donkey anti-sheep IgG. Following incubation with enzyme conjugate and antibody, wells were washed, developed with p-nitrophenyl phosphate substrate, and absorbance was recorded at 405 nm. Concentrations were determined from a standard calibration curve.

### 2.6 Gene expression analysis (RT-qPCR)

Total RNA was extracted from PFC using the Trizol reagent method. Tissue was homogenized in Trizol and RNA was separated using chloroform and precipitated with isopropanol. The RNA pellet was washed with 75% ethanol, air-dried, and resuspended in RNase-free water. Complementary DNA (cDNA) was synthesized from total RNA using the Verso cDNA synthesis kit (Thermo Scientific). Real-time quantitative PCR (RT-qPCR) was performed using the ABI 7900HT system with SYBR Green master mix. The genes assessed included *Nr3c1*, *Il6*, *Oxtr*, *Esr2*, *Casp3*, and *Bdnf*, selected based on their established roles in stress regulation, neuroinflammation, social behavior, and neuroplasticity. The relative expression of target genes was calculated using the 2^–ΔΔ^ ^Ct^ method, with *Gapdh* serving as the endogenous housekeeping gene for normalization. List of primers used is provided in Supplementary Table 1.

### 2.7 Protein expression analysis (immunoblotting)

PFC tissue was homogenized in a lysis buffer containing 8M Urea, 2M Thiourea, 4% CHAPs, and a protease inhibitor cocktail. The lysate was sonicated and centrifuged, and the supernatant containing the total protein was collected. The proteins quantified included BDNF, IL-1β, TNF-α, mGluR2, NMDAR2A, PSD-95, and SHANK3, selected based on their roles in neuroplasticity, inflammatory signaling, and synaptic organization. Protein concentration was determined using the bradford assay with bovine serum albumin (BSA) as a standard. Equal amounts of protein from each sample were separated by SDS-PAGE on polyacrylamide gels and transferred to a polyvinylidene fluoride (PVDF) membrane. Protein bands were visualized using an enhanced chemiluminescence (ECL) detection reagent, and band intensities were quantified using ImageJ software. A list of antibodies used is provided in Supplementary Table 2.

### 2.8 Statistical analysis

Statistical analyses were performed using R software with rstatix and ggpubr packages. Data are generally presented as mean ± standard error of the mean (SEM) or as boxplots representing the median and interquartile range (IQR). The assumption of homogeneity of variance was assessed using Levene’s test. For behavioral and molecular datasets meeting parametric assumptions, a two-way Analysis of Variance (ANOVA) was conducted to evaluate the main effects of Sex and Group and their interaction. Significant effects were followed by Tukey’s Honestly Significant Difference (HSD) *post-hoc* tests to correct for multiple comparisons. For datasets violating normality or homogeneity of variance assumptions, a non-parametric Aligned Rank Transform (ART) ANOVA was employed. *Post-hoc* pairwise comparisons for these non-parametric data were conducted using Dunn’s test with Bonferroni correction. Longitudinal trajectories for sucrose preference and body weight were analyzed using the Friedman test for repeated measures, while endpoint comparisons utilized the Kruskal-Wallis test followed by Dunn’s *post-hoc* test. Specific within-group changes from baseline to endpoint were evaluated using paired Wilcoxon signed-rank tests. All reported *post-hoc p*-values represent multiplicity-adjusted values obtained from Tukey’s HSD or Dunn’s Bonferroni procedures. For protein expression analysis, data were analyzed using a parametric Two-way ANOVA to evaluate the main effects of Sex and Group and their interaction. Upon identifying significant main effects or interactions, specific group differences were evaluated using Fisher’s Protected Least Significant Difference (LSD) *post-hoc* tests to explore sex-specific vulnerabilities. Statistical significance was defined as *p* ≤ 0.05 across all analyses.

## 3 Results

### 3.1 Temporal dynamics of escape counts and latencies in male and female mice

The modified active avoidance test served as the primary measure for validating the induction of the learned helplessness phenotype, defined by the failure to initiate an escape response even when a controllable escape route is made available. Sample sizes were: control (*n* = 8), vicarious (*n* = 9), and demonstrator (*n* = 9). Analysis of total successful escapes (out of 20 trials) showed that control females escaped on average 19.50 times ( ± 0.27 SEM; Supplementary Figure 2), whereas the vicarious group averaged 11.22 escapes ( ± 1.86 SEM) and demonstrators averaged 0.63 escapes ( ± 0.32 SEM). Control males achieved 20.00 escapes ( ± 0.00 SEM; Supplementary Figure 3), while vicarious males averaged 14.56 escapes ( ± 2.05 SEM) and demonstrators averaged 1.00 escape ( ± 0.53 SEM).

**FIGURE 2:**
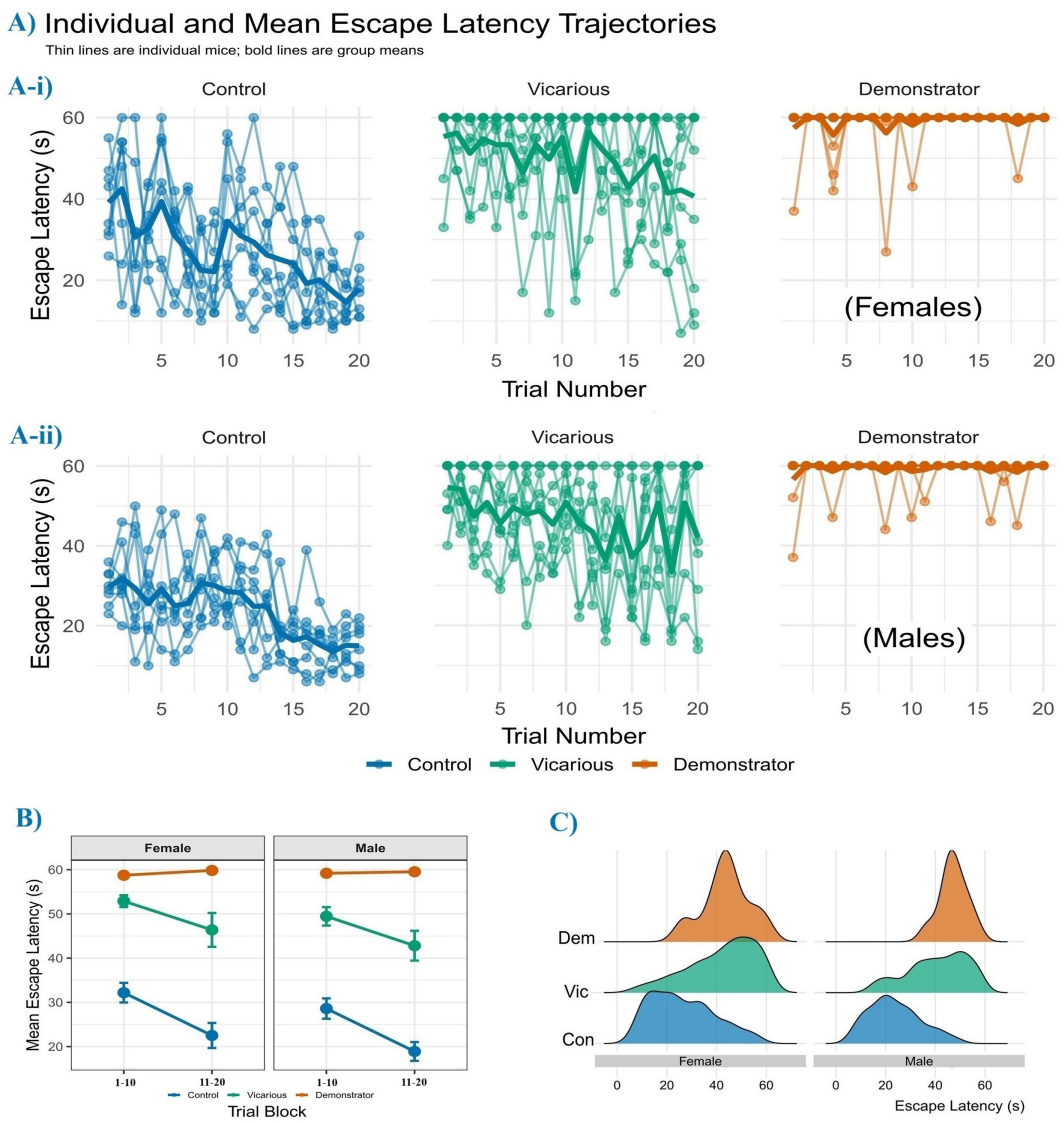
Escape latency learning curves and distributional profiles. **(A)** Visual representation of trial-by-trial escape latency for each individual mouse. Each light-colored line represents the performance trajectory of a single mouse across the 20 trials. The bold, smoothed line in each panel represents the group average learning curve, with the shaded ribbon indicating the 95% confidence interval. This visualization highlights both the overall group trends and the significant individual variability in performance in females **(Ai)** and males **(Aii)**. The y-axis represents the time taken to escape in seconds, with a maximum of 60 s indicating an escape failure. The x-axis represents the trial number. Sample sizes (N) are: control = 8, vicarious = 9, demonstrator = 9. **(B)** Comparison of mean escape latencies during the Acquisition Phase (Trials 1–10) versus the Retention Phase (Trials 11–20). A significant reduction in latency between phases, indicative of successful learning. **(C)** Density distribution of latencies for successful escapes. Control mice exhibit a left-shifted peak indicating frequent and rapid responses (10–30 s). In contrast, the vicarious group displays a flattened, right-shifted distribution, reflecting inefficient escape responses. The demonstrator group profile is negligible due to the scarcity of successful escapes. Sample sizes: control (*n* = 8), demonstrator (*n* = 9), vicarious (*n* = 9).

**FIGURE 3:**
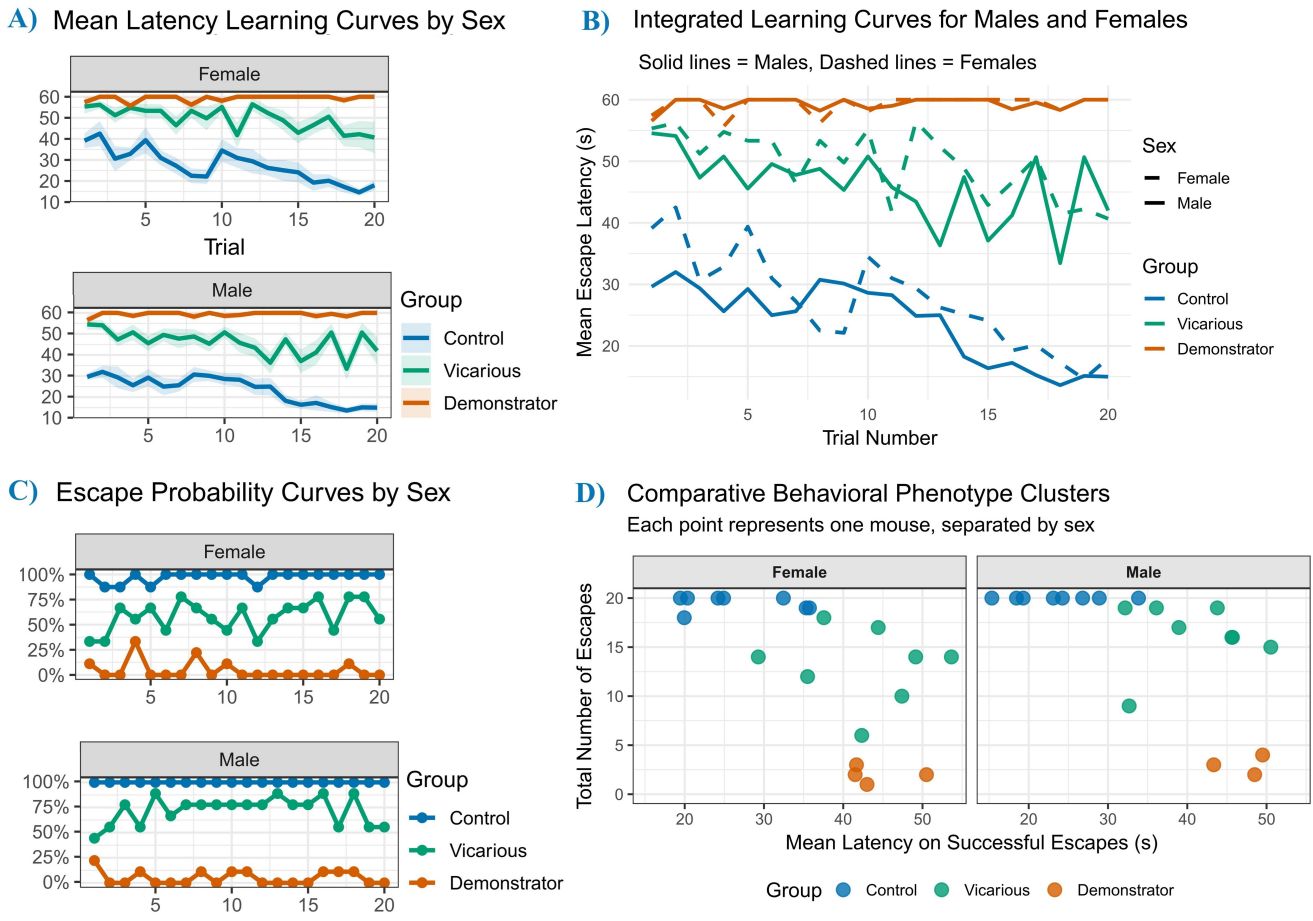
Stress exposure disrupts avoidance performance, response reliability, and escape efficiency. **(A,B)** Overall average escape latency for female and male mice. Control animals exhibited efficient cognitive performance with low mean latencies (Females: 27.4 s; Males: 23.8 s). In contrast, both stress groups demonstrated significant impairment, with demonstrator mice showing the most severe deficits (averaging approximately 59 s) and vicarious mice showing intermediate impairment (Females: 49.6 s; Males: 45.9 s). **(C)** Probability of successful escape. Control mice demonstrated high behavioral reliability, maintaining near-complete escape probability. Demonstrator mice exhibited persistent behavioral inhibition with near-zero success rates, while vicarious mice displayed inconsistent and fluctuating response probabilities (Females:56%; Males:73%), indicating a failure to consolidate a reliable avoidance strategy. **(D)** Efficiency of successful avoidance responses, measured as the mean latency for successful trials. Even when active avoidance was successfully executed, socially stressed mice (vicarious and demonstrator) were significantly slower and less efficient than controls, suggesting impaired cognitive processing speed or motor initiation. Control (*n* = 8), demonstrator (*n* = 9), vicarious (*n* = 9).

Acquisition of active avoidance across 20 trials is shown in Figure 2A. Female (Figure 2Ai) controls showed decreasing escape latencies from 39.1 s ( ± 3.5 SEM) on trial 1 to 18.0 s ( ± 2.4 SEM) by trial 20. Vicarious females maintained higher latencies (55.3 ± 3.2 SEM initially; 40.7 ± 7.5 SEM at Trial 20), whereas demonstrators reached the 60 s cut-off (57.1 ± 2.9 SEM to 60.0 ± 0.0 SEM). A similar pattern was observed in males (Figure 2Aii): control males decreased from 29.6 ± 1.5 SEM to 15.0 ± 1.9 SEM, while vicarious mice remained elevated (54.6 ± 2.4 SEM to 41.9 ± 6.5 SEM) and demonstrators reached the 60 s latency from Trial 10 onward (56.6 ± 2.6 SEM; Trial 20: 60.0 ± 0.0 SEM). Overall mean latency across trials (Figures 3A,B) showed lower latencies in controls compared with stressed groups. Control females averaged 27.4 s ( ± 2.5 SEM), whereas vicarious (49.6 ± 2.5 SEM) and demonstrator females (59.5 ± 0.2 SEM) showed higher latencies. Control males averaged 23.8 s ( ± 2.1 SEM), while vicarious (45.9 ± 2.6 SEM) and demonstrator males (59.4 ± 0.3 SEM) showed higher latencies.

### 3.2 Temporal changes in escape latency: acquisition vs. retention

To confirm the occurrence of learning and minimize the trial-by-trial variability inherent in behavioral testing, we analyzed the change in escape latency by binning the data into two distinct phases: the acquisition phase (First 10 trials) and the retention phase (Last 10 trials) (Figure 2B). Female control mice showed decreased latency from 32.2 s ( ± 2.22 SEM) in the first block to 22.5 s ( ± 2.84 SEM) in the final block. Female demonstrators showed no change, remaining near the 60 s cut-off across both phases (59.1 ± 0.49 SEM to 60.0 ± 0.0 SEM). Female vicarious mice showed higher latencies overall, decreasing from 52.9 s ( ± 1.34 SEM) to 46.4 s (±3.83 SEM). A similar pattern was observed in males. Control males decreased from 28.6 s ( ± 2.30 SEM) during acquisition to 18.9 s ( ± 2.13 SEM) during retention. Male demonstrators remained unchanged (59.2 ± 0.47 SEM to 59.5 ± 0.23 SEM). The male vicarious group showed a slight numerical decrease from 49.5 s ( ± 2.09 SEM) to 42.3 s (±.40 SEM); however, their performance in the final block remained highly variable and significantly slower than controls, indicating a failure to efficiently consolidate the avoidance behavior.

### 3.3 Distribution of successful escape latencies

To verify the efficiency of avoidance responses, we analyzed the distribution of escape latencies (Figure 2C) specifically for trials where mice successfully entered the insulated compartment (i.e., excluding failures to escape, defined as 60 s). Control mice (both male and female) exhibited a left-shifted distribution peak, with the majority of successful escapes occurring at lower latencies (typically between 10 and 30 s). This indicates that when control mice escaped, they did so rapidly and efficiently. In contrast, the vicarious group showed a flatter, right-shifted distribution. Even on trials where vicarious mice successfully avoided the shock, their latencies were generally higher than controls, suggesting delayed decision-making process. Data for demonstrator mice are not shown due to the marked floor effect (near-zero successful escapes), confirming the validity of the inescapable shock protocol.

To complement trial-wise latency analyses, we next evaluated the reliability and efficiency of avoidance performance using probability-based and conditional latency metrics (Figures 3C,D). Analysis of escape probability across trials (Figure 3C) showed efficient performance in control mice of both sexes, whereas demonstrator mice failed to acquire avoidance behavior. Vicarious mice displayed an intermediate but inconsistent response pattern, indicating impaired consolidation of avoidance learning (see Supplementary Results). Analysis of escape efficiency (Figure 3D) revealed that, even on successful trials, both vicarious and demonstrator mice exhibited significantly prolonged escape latencies compared to controls, reflecting reduced response efficiency across sexes (detailed in Supplementary Results).

### 3.4 Vicarious and direct trauma impair active avoidance learning

**FIGURE 4:**
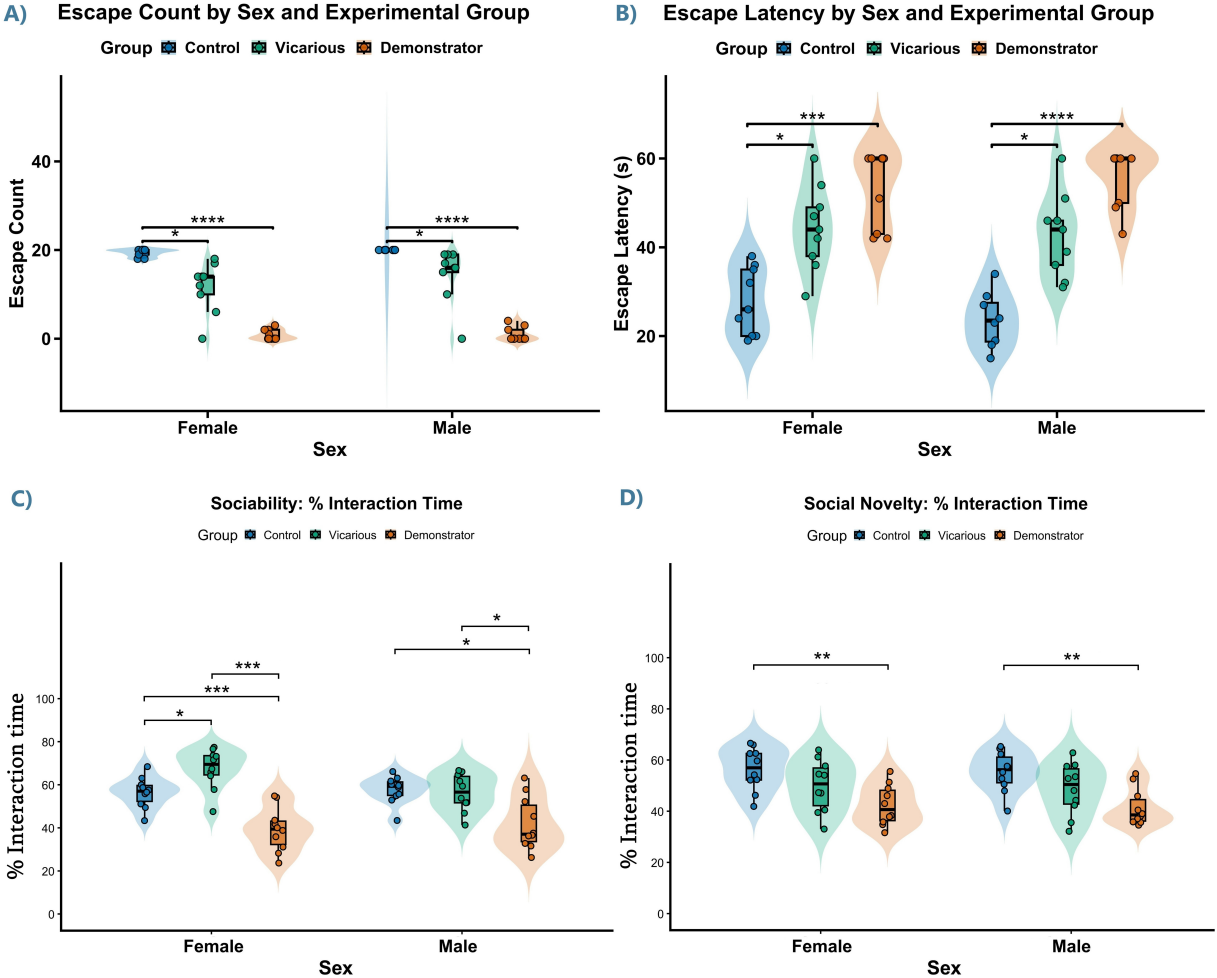
Vicarious and direct social trauma impair active avoidance learning and differentially induce social interaction deficits. **(A)** Total Successful Escapes: In both females and males, control animals exhibited significantly higher escape counts than both vicarious (*p* < 0.05) and demonstrator (*p* < 0.0001) groups. **(B)** Escape Latency: Analysis of escape latency revealed a significant main effect of Group with no interaction by Sex. Both female and male controls displayed significantly shorter latencies compared to vicarious (*p* < 0.05) and demonstrator (Females: *p* < 0.001, Males: *p* < 0.0001) groups. Data represent Mean ± SEM. **(C,D)** Social interaction deficits following VLH exposure. **(C)** Sociability (% interaction time). Sociability was assessed as the percentage of time spent interacting with a conspecific versus an empty chamber. *Post-hoc* comparisons showed that in females, vicarious (*p* = 0.027) group increased sociability and demonstrator (*p* = 0.001) groups exhibited reduced sociability compared with controls. In males, the demonstrator group showed reduced sociability compared with controls (*p* = 0.021) and vicarious animals (*p* = 0.034), whereas control vs. vicarious males were not different (*p* = 1.000). **(D)** Social novelty preference (% interaction time). Social novelty was calculated as the percentage of interaction time with a novel versus a familiar conspecific. Post-hoc analysis showed impaired novelty preference in demonstrator mice in both males (*p* = 0.003) and females (*p* = 0.006) relative to controls, while vicarious groups showed no significant deficits. Statistical significance was determined using Two-Way ART ANOVA followed by pairwise Wilcoxon tests with Bonferroni correction (*p ≤ 0.05, **p ≤ 0.01, ***p ≤ 0.001, ****p ≤ 0.0001).

Analysis of the total number of successful escapes revealed no significant interaction between Sex and Group [*F*(2, 47) = 1.86, *p* = 0.168], indicating that the impact of the experimental conditions was consistent across both males and females. However, a highly significant main effect of Group was observed (*p* < 0.001). To elucidate the specific group differences contributing to this effect, pairwise comparisons were conducted within each sex. Control females (Figure 4A) exhibited significantly higher escape counts compared to both the vicarious group (*p* = 0.031) and the demonstrator group (*p* < 0.0001). The difference between the vicarious and demonstrator groups did not reach statistical significance (*p* = 0.115). A parallel pattern was observed in the male cohort. Control males demonstrated significantly higher escape counts than both the vicarious (*p* = 0.034) and demonstrator (*p* < 0.0001) groups. Similar to females, the difference between the vicarious and demonstrator groups was not statistically significant (*p* = 0.088). These findings confirm that both vicarious and direct social stress exposure significantly reduce the frequency of successful escape behaviors relative to controls in both sexes.

The analysis of escape latency further corroborated the behavioral deficits induced by stress (Figure 4B). The ART ANOVA revealed no significant main effect of Sex [*F*(1, 47) = 0.16, *p* = 0.690] and no significant Sex × Group interaction [*F*(2, 47) = 1.38, *p* = 0.262], suggesting that basal escape speeds and the response to stress were comparable between males and females. A significant main effect of Group was identified [*F*(2, 47) = 53.16, *p* < 0.001]. Pairwise comparisons were performed. Control female mice displayed significantly shorter escape latencies compared to both the vicarious (*p* = 0.022) and demonstrator (*p* < 0.001) groups. No significant difference was found between the vicarious and demonstrator groups (*p* = 0.508). Consistent with the female data, control males exhibited significantly shorter latencies than both the vicarious (*p* = 0.033) and demonstrator (*p* < 0.0001) groups. The difference between the vicarious and demonstrator groups was not statistically significant (*p* = 0.199).

### 3.5 Vicarious and direct stress differentially impair sociability and social novelty preference

To assess the impact of stress exposure on social behavior and empathy associated emotional response, mice were evaluated for sociability (*n* = 10 for all groups) and preference for social novelty (Figures 4C,D). Analysis of sociability interaction time revealed a significant main effect of Group [*F*(2, 54) = 26.16, *p* < 0.0001] along with a significant Sex × Group interaction [*F*(2, 54) = 3.36, *p* = 0.0421]. No significant main effect of Sex was observed [*F*(1, 54) = 1.09, *p* = 0.301]. *Post hoc* analysis in females revealed that vicarious (*p* = 0.027) group significantly increased while demonstrator mice (*p* = 0.001) significantly reduced sociability compared to controls. In contrast, males exhibited a selective deficit restricted to the demonstrator group, which showed reduced sociability relative to both control (*p* = 0.021) and vicarious animals (*p* = 0.034), while vicarious males remained comparable to controls (*p* = 1.000). Analysis of social novelty preference revealed a significant main effect of Group [*F*(2, 54) = 13.20, *p* < 0.001] without significant effects of Sex or interaction, suggesting a conserved deficit across sexes. *Post-hoc* comparisons showed that demonstrator mice displayed impaired novelty preference in both males (*p* = 0.003) and females (*p* = 0.006) relative to controls, whereas vicarious groups did not show any significant alterations. Findings indicate that vicarious stress primarily disrupts sociability in a sex-dependent manner, while direct trauma produces broader impairments in social cognition across both sexes.

### 3.6 Behavioral deficits induced by the vicarious learned helplessness paradigm

**FIGURE 5:**
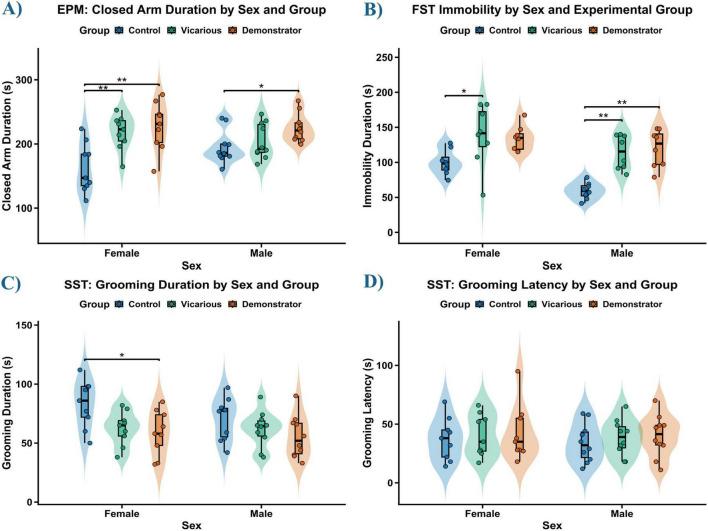
Vicarious and direct social stress induce anxiety-like behavior, behavioral despair, and anhedonia in a sex-dependent manner. **(A)** Duration of time spent in the closed arms of the Elevated Plus Maze (EPM): Social stress exposure significantly increased anxiety-like behavior (main effect of Group, *p* < 0.001). In females, both vicarious and demonstrator groups spent significantly more time in the closed arms compared to controls. In males, this anxiogenic effect was specific to direct stress (demonstrator group), with vicarious males showing no significant difference from controls. **(B)** Immobility duration in the Forced Swim Test (FST): Male mice exhibited increase in depressive-like immobility following both vicarious and direct stress exposure. In females, the vicarious group showed significantly increased immobility, while the demonstrator group displayed a trend toward significance (*p* = 0.054). **(C)** Total grooming duration in the Sucrose Splash Test (SST): Female demonstrator mice displayed significantly reduced grooming time, indicative of self-care deficits. Male mice remained resilient, showing no significant changes in grooming duration. **(D)** Latency to initiate grooming in the SST was unaltered across all groups and sexes. Data represent Mean ± SEM. Significant differences relative to control are indicated (*p ≤ 0.05, **p ≤ 0.01).

To evaluate the impact of vicarious and direct social stress on anxiety-like behavior, we analyzed the duration of time spent in the closed arms of the EPM (Figure 5A). Behavioral analyses were conducted with group sizes ranging from *n* = 9 to 10. The analysis revealed a highly significant main effect of Group [*F*(2, 51) = 12.45, *p* < 0.001], indicating that stress exposure increased anxiety levels. There was no significant main effect of Sex [*F*(1, 51) = 0.53, *p* = 0.470]. The Sex × Group interaction approached but did not reach statistical significance [*F*(2, 51) = 2.82, *p* = 0.069]. In the female cohort, both stress groups exhibited increased anxiety. The vicarious group spent significantly more time in the closed arms compared to controls (*p* = 0.006). Similarly, the demonstrator group showed a significant increase in closed arm duration relative to controls (*p* = 0.002). No significant difference was observed between the vicarious and demonstrator females (*p* = 0.840). In the male cohort, the anxiety response was specific to direct stress. The demonstrator group spent significantly more time in the closed arms than controls (*p* = 0.032). However, unlike females, the vicarious group did not differ significantly from controls (*p* = 0.674).

Depressive-like behavior was assessed by measuring the duration of immobility in the FST (Figure 5B). Due to violation of the assumption of homogeneity of variance (*p* < 0.05), a non-parametric ART ANOVA was utilized. The analysis revealed a significant main effect of Sex [*F*(1, 47) = 17.51, *p* < 0.001] and a highly significant main effect of Group [*F*(2, 47) = 13.91, *p* < 0.001]. There was no significant interaction between Sex and Group (*p* = 0.730). *Post-hoc* comparisons (Dunn’s test with Bonferroni correction) were performed to clarify group differences. In female mice, the vicarious group displayed a significant increase in immobility compared to controls (*p* = 0.019). The demonstrator group showed a trend toward increased immobility, though it narrowly missed statistical significance (*p* = 0.054). In male mice, within-sex comparisons revealed strong depressive-like phenotype. Both the vicarious (*p* = 0.004) and demonstrator (*p* = 0.001) groups exhibited significantly higher immobility durations compared to controls.

To assess self-care behavior, we analyzed grooming duration in the Sucrose Splash Test (SST) (Figure 5C). A significant main effect of Group was observed [*F*(2, 51) = 6.44, *p* = 0.003], while the main effect of Sex (*p* = 0.232) and the Interaction (*p* = 0.535) were not significant. *Post-hoc* analysis revealed that the behavioral deficits were predominantly driven by the female cohort. Female demonstrator mice spent significantly less time grooming compared to controls (*p* = 0.021), indicating a stress-induced deficit in self-care. The female vicarious group showed a strong trend toward reduced grooming (*p* = 0.055). In contrast, male mice showed no statistically significant differences between any groups (all *p* > 0.05). We also analyzed the latency to initiate grooming (Figure 5D). The two-way ANOVA indicated no significant effects of Sex (*p* = 0.611), Group (*p* = 0.633), or their interaction (*p* = 0.993).

### 3.7 Impact of vicarious learned helplessness on anhedonia and body weight

**FIGURE 6:**
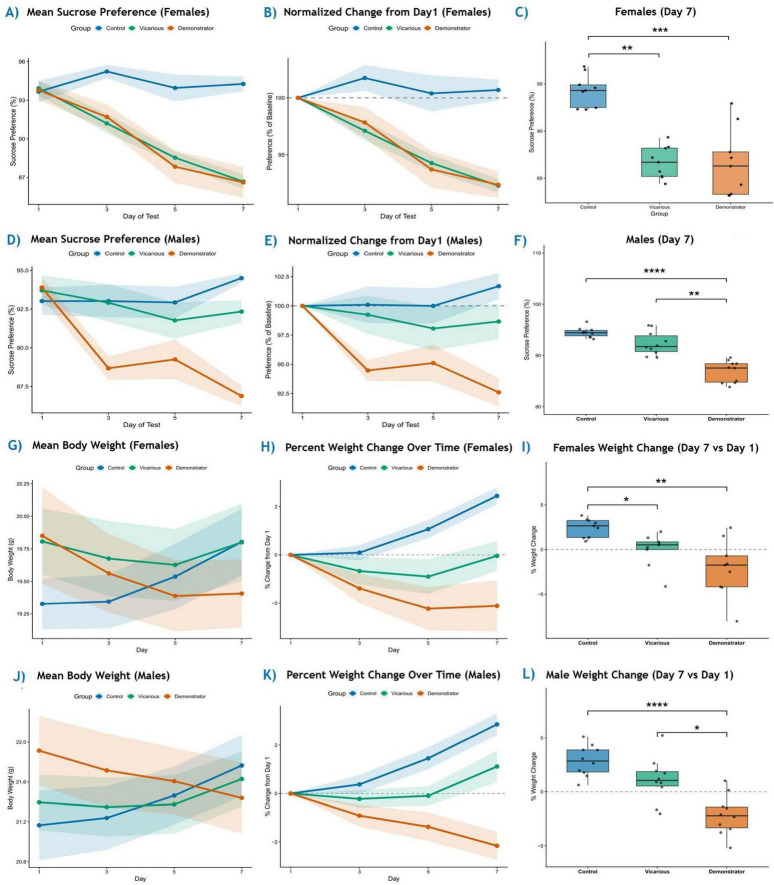
Vicarious learned helplessness induces anhedonia-like behavior and alters body weight trajectories. **(A–C)** In females (*n* = 9 per group), longitudinal sucrose preference analysis using Friedman tests showed stable preference in controls [χ^2^(3) = 1.98, *p* = 0.577], whereas vicarious [χ^2^(3) = 23.5, *p* < 0.001] and demonstrator groups [χ^2^(3) = 18.2, *p* < 0.001] exhibited significant fluctuations **(A)**. Normalization to individual day 1 baselines revealed a progressive decline in demonstrator (*p* = 0.002) and vicarious females (*p* = 0.002), with no change in controls (*p* = 0.694) **(B)**. Day 7 endpoint analysis demonstrated a significant group effect [*H*(2) = 16.2, *p* < 0.001]; Dunn’s post hoc tests showed reduced preference in demonstrator (*p* < 0.001) and vicarious females (*p* = 0.003) versus controls, with no difference between stressed groups (*p* > 0.99) **(C)**. **(D–F)** In males (*n* = 10 per group), sucrose preference remained stable in controls [χ^2^(3) = 1.08, *p* = 0.782] and vicarious animals [χ^2^(3) = 1.56, *p* = 0.668], whereas demonstrator males showed significant downregulation [χ^2^(3) = 18.3, *p* < 0.001] **(D)**. Baseline-normalized and paired Wilcoxon analyses confirmed a significant decline only in demonstrators (*p* = 0.001), with no change in controls (*p* = 0.903) or vicarious males (*p* = 0.216) **(E)**. Endpoint analysis revealed a significant group effect [*H*(2) = 21.4, *p* < 0.001], with demonstrators differing from controls (*p* < 0.0001) and vicarious males (*p* = 0.007) **(F)**. **(G–L)** Body Weight Dynamics. Absolute body weight trajectories in females **(G)** and males **(J)** show significant growth in controls (Friedman test, *p* < 0.001) that is absent in experimental groups. Percent weight change trajectories **(H,K)** illustrate this stagnation. **(I)** Total weight change at day 7 in females was significantly lower in both vicarious (*p* = 0.027) and demonstrator (*p* = 0.001) groups compared to controls. **(L)** In males, the demonstrator group exhibited significant weight loss compared to both control (*p* < 0.0001) and vicarious groups (*p* = 0.036). Data presented as mean ± SEM (trajectories) or median with interquartile range (boxplots). Statistics derived from Kruskal-Wallis tests followed by Dunn’s *post-hoc* comparisons. Significant differences relative to control are indicated (*p ≤ 0.05, **p ≤ 0.01, ***p ≤ 0.001, ****p ≤ 0.0001).

Sucrose preference was monitored longitudinally. In females (*n* = 9) (Figure 6A) a Friedman test for repeated measures showed that preference remained stable in controls across testing days [χ^2^(3) = 1.98, *p* = 0.577]. In contrast, both vicarious [χ^2^(3) = 23.5, *p* < 0.001] and demonstrators [χ^2^(3) = 18.2, *p* < 0.001] exhibited significant fluctuations. Normalization to individual mouse day 1 baselines (Figure 6B) revealed a progressive decline in demonstrator (*p* = 0.002) and vicarious females (*p* = 0.002), whereas controls remained unchanged (*p* = 0.694). At the day 7 endpoint (Figure 6C), a Kruskal-Wallis test demonstrated a significant main effect of group [*H*(2) = 16.2, *p* < 0.001]. *Post-hoc* Dunn’s tests revealed that demonstrator females displayed significantly lower sucrose preference compared to controls (*p* < 0.001). Notably, vicarious females also differed significantly from controls (*p* = 0.003), and did not differ from demonstrators (*p* > 0.99).

Longitudinal analysis (Figure 6D) revealed stable sucrose preference across time in control [χ^2^(3) = 1.08, *p* = 0.782] and vicarious males [χ^2^(3) = 1.56, *p* = 0.668], whereas demonstrator males showed significant downregulation [χ^2^(3) = 18.3, *p* < 0.001] (*n* = 10 per group). Normalization to baseline (Figure 6E) and paired Wilcoxon tests indicated a significant reduction in preference from days 1 to 7 exclusively in demonstrator males (*p* = 0.001), with no significant decline observed in control (*p* = 0.903) or vicarious males (*p* = 0.216). Endpoint analysis on day 7 (Figure 6F) revealed a significant group effect [*H*(2) = 21.4, *p* < 0.001]. Post*-hoc* comparisons demonstrated that demonstrator males exhibited significantly lower sucrose preference relative to both controls (*p* < 0.0001) and vicarious (*p* = 0.007).

We further examined body weight as a physiological correlate of stress. In female control, body weight trajectories (Figure 6G) indicated significant increase over time [χ^2^(3) = 18.5, *p* < 0.001] and a significant gain from day 1 to day 7 (*p* = 0.004) (Figure 6H). Conversely, both vicarious and demonstrator females failed to show significant growth over time (*p* > 0.20 for both) and their day 7 weight did not differ significantly from baseline (Vicarious: *p* = 0.624; Demonstrator: *p* = 0.074). Analysis of the total percent weight change at day 7 showed a significant group difference [*H*(2) = 13.4, *p* = 0.0012]. Dunn’s *post-hoc* tests revealed that control females gained significantly more weight than both vicarious observers (*p* = 0.027) and demonstrators (*p* = 0.0012) (Figure 6I).

In males, weight trajectories (Figure 6J) showed diverging patterns. Control males exhibited significant growth over time [χ^2^(3) = 23.9, *p* < 0.001] and a significant gain from baseline to day 7 (*p* = 0.002). Vicarious males maintained stable weight [χ^2^(3) = 6.96, *p* = 0.073; day 1 vs. D7: *p* = 0.185]. Notably, demonstrator males showed a significant change over time [χ^2^(3) = 10.4, *p* = 0.015] characterized by significant weight loss from baseline to day 7 (*p* = 0.010). These distinct patterns are evident in the percent change trajectories (Figure 6K). Endpoint analysis of day 7 percent weight change (Figure 6L) revealed a significant main effect [*H*(2) = 17.8, *p* < 0.001]. *Post-hoc* tests confirmed that demonstrator males had significantly lower weight change outcomes compared to both controls (*p* < 0.0001) and vicarious males (*p* = 0.036). There was no significant difference in endpoint weight change between control and vicarious males (*p* = 0.28). Individual mouse trajectories are represented in Supplementary Figure 4.

### 3.8 Physiological stress response: plasma corticosterone levels

**FIGURE 7:**
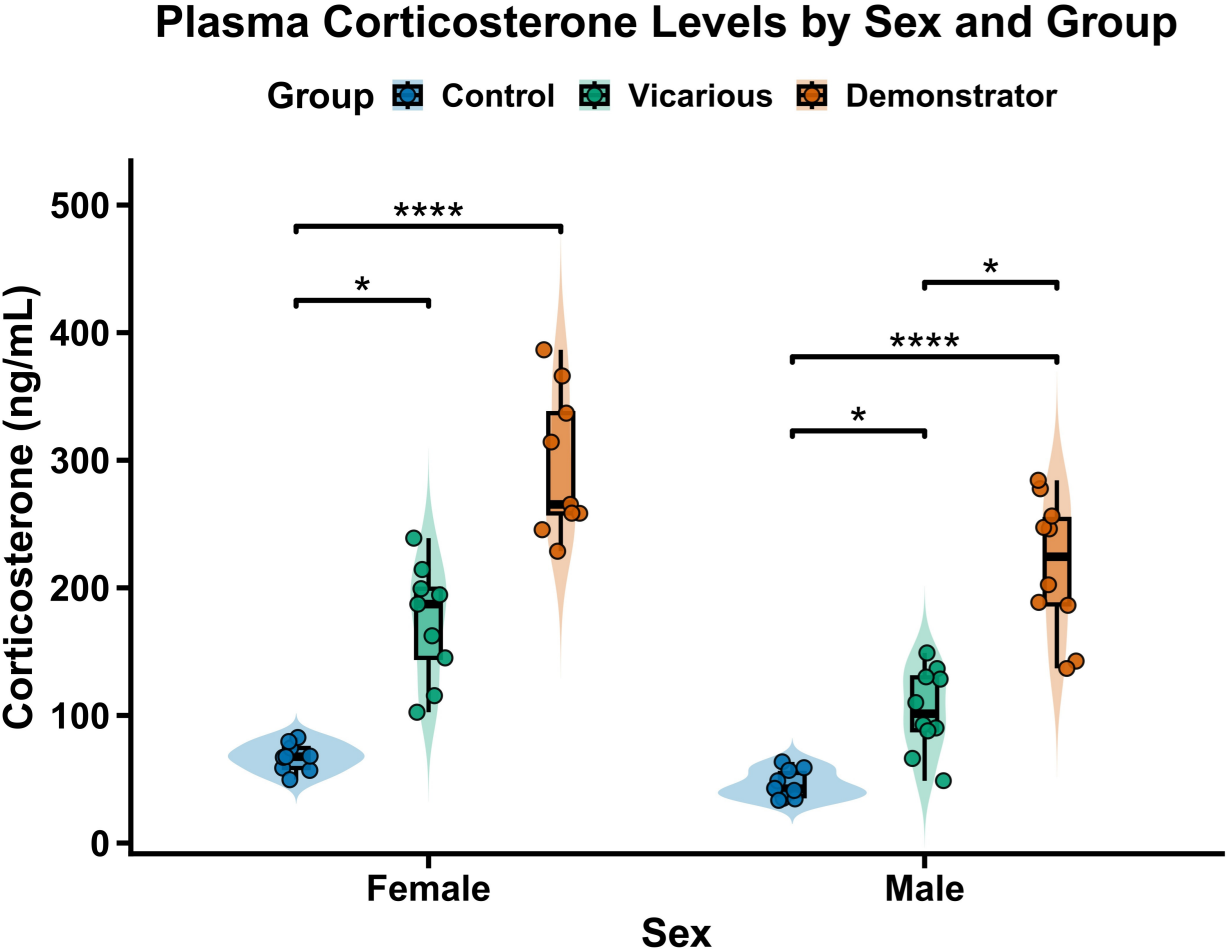
Effect of vicarious learned helplessness on plasma corticosterone levels. Raincloud plots illustrate the distribution of plasma corticosterone concentration (ng/mL) in male and female mice across control, vicarious, and demonstrator groups (n = 9-10 per group). The plots combine a probability density distribution (violin), boxplots representing the median and interquartile range (IQR), and individual data points. Data were analyzed using a non-parametric ART ANOVA followed by Dunn’s post-hoc test with Bonferroni correction. In males, both vicarious and demonstrator groups showed significantly elevated corticosterone compared to controls, with demonstrators exhibiting a further significant increase over the vicarious group. In females, both vicarious and demonstrator groups showed significantly higher corticosterone levels compared to controls, though they did not differ significantly from each other. Asterisks indicate statistically significant differences (*p ≤ 0.05, ****p ≤ 0.0001).

To evaluate the physiological impact of the VLH protocol, plasma corticosterone (CORT) levels were quantified in male and female mice (Figure 7). Data were analyzed using non-parametric ART ANOVA. The analysis revealed a highly significant main effect of Group [*F*(2, 51) = 159.29, *p* < 0.001], confirming that the experimental procedures induced a strong stress response. A significant main effect of Sex was also observed [*F*(1, 51) = 32.68, *p* < 0.001], reflecting basal differences in corticosterone levels between sexes. The Sex by Group interaction approached but did not reach statistical significance [*F*(2, 51) = 2.86, *p* = 0.067], suggesting that while the overall pattern of stress response was similar, there were subtle sex-dependent variations in the magnitude of the effect.

To delineate specific group differences, pairwise comparisons were conducted using Dunn’s test with Bonferroni correction. In the female cohort (*n* = 9 per group), exposure to stress resulted in significant physiological activation. Both the vicarious (*p* = 0.045) and demonstrator (*p* < 0.0001) groups displayed significantly higher corticosterone levels compared to controls. However, the difference between the vicarious and demonstrator groups in females did not reach statistical significance (*p* = 0.057), although a strong trend toward higher levels in demonstrators was evident. In males (*n* = 10 per group), the vicarious group exhibited significantly elevated corticosterone compared to the control group (*p* = 0.044). The male demonstrator group showed the highest stress response, with corticosterone levels significantly exceeding both the control group (*p* < 0.0001) and the vicarious group (*p* = 0.036).

### 3.9 Vicarious stress induces transcriptional alterations in the prefrontal cortex

**FIGURE 8:**
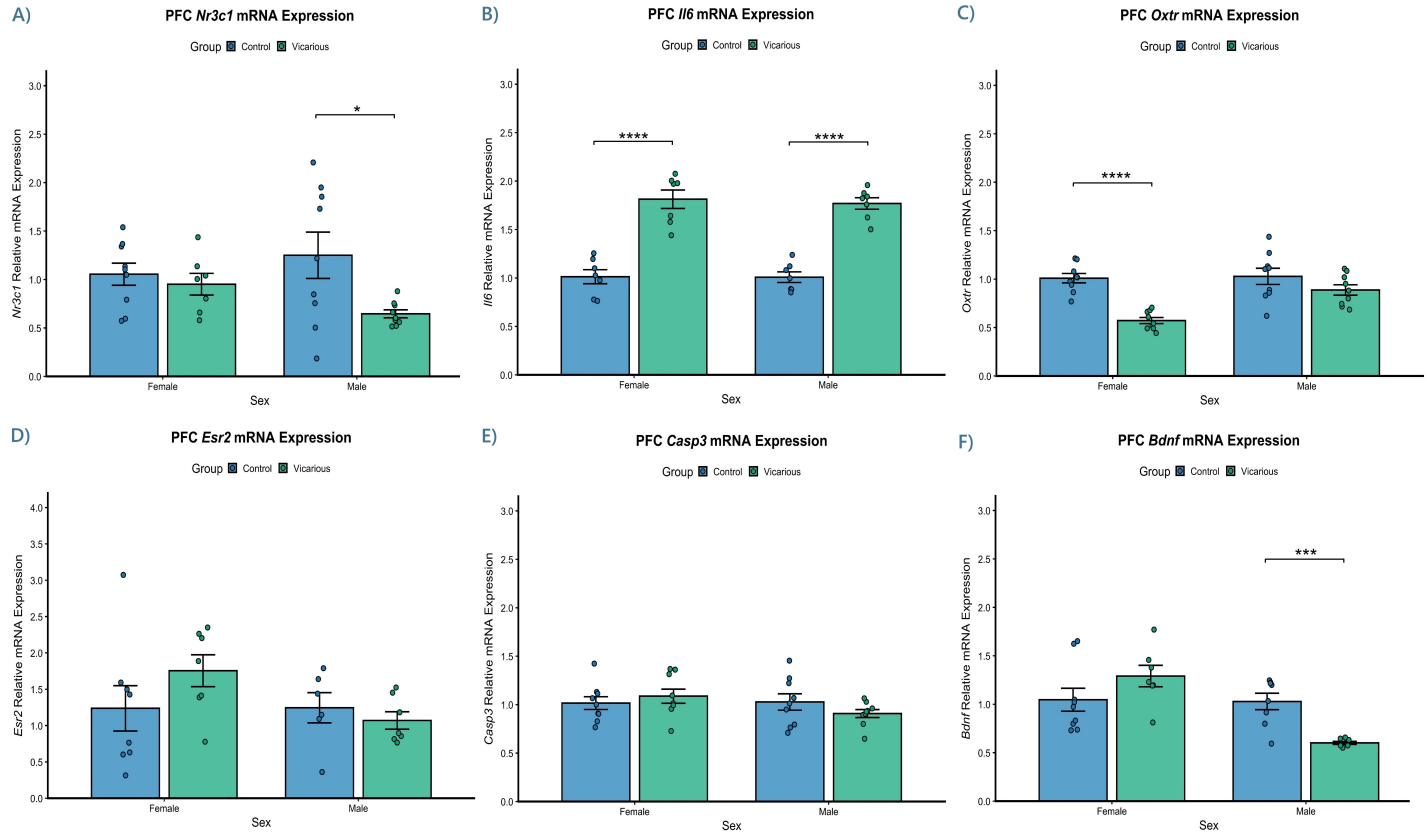
Vicarious stress induces sex-specific transcriptional alterations in the prefrontal cortex. Relative mRNA expression of **(A)** *Nr3c1*, **(B)** *Il6*, **(C)** *Oxtr*, (D) *Esr2*, **(E)** *Casp3*, and **(F)** *Bdnf* was quantified using qPCR. Data are normalized to sex-matched controls (control mean = 1.0) and presented as mean ± SEM. Statistical analysis was performed using two-way ANOVA followed by Tukey’s HSD *post-hoc* test. **(A)** *Nr3c1* was significantly downregulated in vicarious males but unchanged in females. **(B)** *Il6* was robustly upregulated in both male and female vicarious groups. **(C)** *Oxtr* expression was significantly reduced specifically in vicarious females. No significant differences were observed for **(D)** *Esr2* or **(E)** *Casp3*. **(F)** *Bdnf* levels were significantly decreased in vicarious males, with no significant change in females. Significance is denoted as (*p ≤ 0.05, ***p ≤ 0.001, ****p ≤ 0.0001).

The primary objective of the present study was to isolate the neurobiological consequences of purely psychological stress, independent of physical trauma. Therefore, our molecular analyses were centered on the vicarious group, which experienced stress exclusively through social observation of conspecific distress. We quantified the mRNA expression of key genes associated with stress regulation, inflammation, social bonding, and neuroplasticity in the PFC (*n* = 8–10 per group). The glucocorticoid receptor (*Nr3c1*) is a critical regulator of the HPA axis negative feedback loop. A parametric two-way ANOVA revealed a significant main effect of Group [*F*(1, 30) = 5.31, *p* = 0.028], indicating that vicarious stress altered *Nr3c1* expression (Figure 8A). *Post-hoc* analysis using Tukey’s HSD revealed a sex-specific susceptibility: male vicarious mice exhibited a significant downregulation of *Nr3c1* mRNA compared to male controls (*p* = 0.024). In contrast, female vicarious mice maintained *Nr3c1* expression levels comparable to controls (*p* = 0.535), suggesting a potential resilience in the female HPA axis regulation under these conditions. Given the link between stress and inflammation, we examined Interleukin-6 (*Il6*) expression (Figure 8B). We observed a highly significant main effect of Group [*F*(1, 24) = 117.24, *p* < 0.001], with no significant main effect of Sex or interaction. *Post-hoc* analysis confirmed a significant upregulation of *Il6* mRNA in both the male vicarious group (*p* < 0.0001) and the female vicarious group (*p* < 0.0001) compared to their respective controls. This indicates that vicarious stress triggers neuroinflammatory response in the PFC that is conserved across sexes. We investigated receptors critical for social behavior. For the Oxytocin receptor (*Oxtr*, Figure 8C), a significant Sex × Group interaction was observed [*F*(1, 32) = 6.99, *p* = 0.013]. *Post-hoc* analysis revealed that while male mice showed no significant change (*p* = 0.176), female vicarious mice exhibited a significant downregulation of *Oxtr* expression compared to controls (*p* < 0.0001). Conversely, the expression of Estrogen Receptor 2 (*Esr2*, Figure 8D) remained stable, with no significant effects of Group (*p* = 0.363) or Sex (*p* = 0.226) detected. To assess cellular death and plasticity, we measured Caspase 3 (*Casp3*) and Brain-derived neurotrophic factor (*Bdnf*). For *Casp3* (Figure 8E), no significant main effects of Group (*p* = 0.731) or Sex (*p* = 0.170) were found, indicating the absence of overt apoptotic signaling. However, *Bdnf* expression (Figure 8F) showed a significant Sex × Group interaction [*F*(1, 27) = 12.21, *p* = 0.002]. Male vicarious mice exhibited a significant downregulation of *Bdnf* compared to controls (*p* < 0.001), whereas females showed no significant reduction (*p* = 0.163).

### 3.10 Vicarious stress induces sex-specific alterations in prefrontal protein expression

**FIGURE 9:**
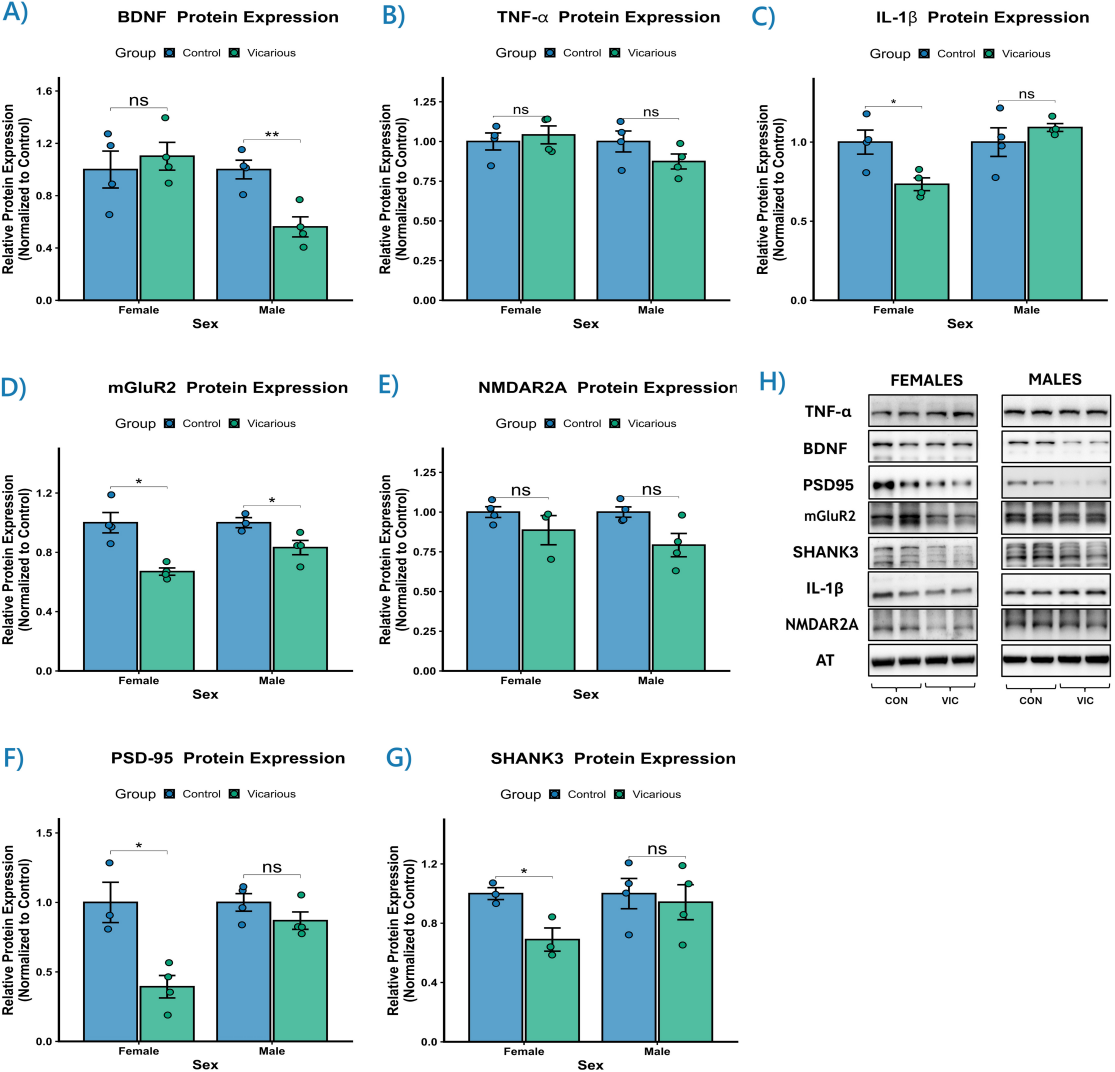
Vicarious stress induces sexually dimorphic alterations in prefrontal protein expression. **(A–G)** Densitometric quantification and **(H)** representative immunoblots of neuroplasticity, inflammatory, and synaptic markers in the prefrontal cortex (control vs. vicarious). Data were normalized to sex-matched controls (control mean = 1.0) and are presented as mean ± SEM. **(A)** BDNF protein levels were significantly downregulated in vicarious males (**p < 0.01) but remained unchanged in females. **(B)** TNF-α expression remained unaltered across all groups. **(C)** Mature IL-1β was significantly reduced specifically in vicarious females (*p < 0.05). **(D)** mGluR2 expression was significantly downregulated in both female and male vicarious groups (*p < 0.05). **(E)** NMDAR2A displayed a significant main effect of Group, driven by a trend toward reduction in males. **(F,G)** The scaffolding proteins PSD-95 and SHANK3 exhibited female-specific vulnerability, with significant downregulation observed only in vicarious females (*p < 0.05). **(H)** Representative bands for target proteins and the loading control (α-Tubulin). Statistical analysis was performed using a two-way ANOVA followed by Fisher’s Protected LSD. Significance is denoted as *p ≤ 0.05, **p ≤ 0.01.

To determine whether the transcriptional alterations observed in our gene expression analysis translated to functional changes at the translational level, we quantified the protein expression of key neuroplasticity-related, inflammatory, and synaptic markers in the PFC (*n* = 4 per group). We first examined neurotrophic support and inflammatory signaling. Analysis of BDNF, a critical modulator of synaptic plasticity, revealed a significant Sex × Group interaction [*F*(1, 12) = 6.92, *p* = 0.022] (Figure 9A). *Post-hoc* pairwise comparisons using Fisher’s Protected LSD demonstrated that this effect was driven entirely by the male cohort. Male vicarious mice exhibited a significant downregulation of BDNF protein compared to controls (*p* = 0.006), whereas female mice remained resilient, showing no significant deviation from control levels (*p* = 0.588). In parallel, we assessed the inflammatory profile by quantifying Interleukin-1 beta (IL-1β) and Tumor Necrosis Factor-alpha (TNF-α). Interestingly, the expression of TNF-α remained stable across all groups, with no significant main effects or interactions (*p* > 0.05) (Figure 9B), suggesting that the modulation of neuroimmune signaling by vicarious stress is selective rather than global. For IL-1β, a significant Sex × Group interaction was observed [*F*(1, 12) = 7.92, *p* = 0.016] (Figure 9C). In a direct contrast to the BDNF findings, pairwise comparisons revealed that female vicarious mice displayed a significant downregulation of mature IL-1β protein (*p* = 0.021), while males showed no significant change (*p* = 0.364).

Next, we investigated the integrity of glutamatergic signaling by assessing receptor subunits. The metabotropic glutamate receptor 2 (mGluR2) emerged as a shared target of stress pathology (Figure 9D). A significant main effect of Group was found [*F*(1, 11) = 25.47, *p* < 0.001], with no interaction. Pairwise comparisons confirmed that vicarious stress significantly downregulated mGluR2 protein levels in both females (*p* = 0.012) and males (*p* = 0.037). Conversely, the ionotropic NMDA receptor subunit 2A (NMDAR2A) displayed a significant main effect of Group [*F*(1, 11) = 7.42, *p* = 0.020] (Figure 9E) that was primarily driven by the male cohort. Males showed a strong trend toward downregulation (*p* = 0.059), however it did not reach statistical significance, while females exhibited control like expression levels (*p* = 0.341). Finally, we evaluated postsynaptic structural integrity by quantifying the scaffolding proteins PSD-95 and SHANK3. Both markers revealed a specific vulnerability in females. For PSD-95, a significant Sex × Group interaction was detected [*F*(1, 11) = 7.66, *p* = 0.018] (Figure 9F). *Post-hoc* analysis showed a significant reduction in PSD-95 levels in female vicarious mice (*p* = 0.032), but not in males (*p* = 0.191). Similarly, SHANK3 expression showed a significant main effect of Group [*F*(1, 10) = 5.61, *p* = 0.039] (Figure 9G). Exploratory pairwise comparisons confirmed that this reduction was significant in females (*p* = 0.038) but absent in males (*p* = 0.721). The data depict that while mGluR2 downregulation is a common consequence of vicarious stress, males primarily exhibit deficits in neurotrophic support (BDNF) and excitatory receptor subunits, whereas females exhibit pronounced deficits in postsynaptic scaffolding architecture and specific inflammatory signaling.

## 4 Discussion

The validity of the VLH model rests on the capacity of rodents to experience “social contagion” or forms of empathy. Empathy, often considered a uniquely human trait, has deep evolutionary roots. Seminal work by Langford and colleagues (Langford et al., 2006) demonstrated that mice exhibit hypersensitivity to pain when observing a cagemate in distress, a phenomenon termed “emotional contagion.” This “social modulation of pain” was dependent on visual cues and social familiarity, suggesting that rodents possess the neural circuitry to represent the affective state of a conspecific. Subsequent studies by Pisansky and colleagues established that fear could be transmitted visually (Pisansky et al., 2017). The VLH paradigm extends these findings from transient states of pain or fear to a chronic, maladaptive state of depression. Our data suggests that the “contagion” is not merely a momentary emotional resonance but a transfer of stress physiology. The observer mouse internalizes the demonstrator’s hopelessness. This suggests for a “shared representations” theory of empathy, where observing a state activates similar neural representations as experiencing it. In the context of chronic stress, this shared representation becomes a shared pathology, recruiting the HPA axis and driving neuroplastic changes in the PFC that persist after the observation has ended.

Historically, animal models of depression have relied on direct physical stressors, such as electric shocks in the learned helplessness paradigm or physical aggression in social defeat models (Golden et al., 2011; Smith et al., 2023; Patel et al., 2026). While effective, these models confound physical pain with psychological distress. Our VLH paradigm isolates the psychological component, demonstrating that visual, auditory, and olfactory cues of conspecific suffering are sufficient to drive a helplessness state. Vicarious stress paradigms engage a distributed cortico-limbic network that integrates emotional contagion, social cognition, and executive appraisal. Evidence from observational fear and vicarious defeat models indicates that regions such as the anterior cingulate cortex (ACC), insular cortex, amygdala, and hippocampus contribute to affective resonance and salience processing (Patel et al., 2025b). Within the context of learned helplessness, dysfunction of prefrontal circuitry is strongly linked to impaired action-outcome processing and deficits in adaptive coping, making it a mechanistically relevant target for investigating vicariously acquired helplessness states. In the present study, we therefore focused our molecular analyses on the PFC as this region represents a convergence point where socially transmitted stress signals translate into executive dysfunction and long-lasting neuroplastic adaptations.

In the Modified Active Avoidance task, which serves as the primary measure for validating learned helplessness, vicarious mice showed a significant reduction in escape success and increased latency. This failure to initiate an escape response, even when a controllable route is available, signifies that the organism has internalized a lack of control, a core cognitive deficit in depression. Our data revealed that while control animals improved their escape efficiency over trials, vicarious mice displayed flattened learning curves. Notably, even when vicarious mice successfully escaped, they did so with significantly slower reaction times than controls, suggesting impaired cognitive processing speed or psychomotor retardation. Avoidance performance was reduced but not completely abolished, indicating that VLH captures a graded form of helplessness rather than a classical all-or-none phenotype.

The differential effects of vicarious stress on sociability and social novelty provide important insight into the empathy-related nature of the VLH paradigm. Notably, female vicarious mice exhibited increased sociability diverging from classical stress models that predict social withdrawal (Harris et al., 2018; Zhai et al., 2024). This pattern may reflect increased emotional contagion or empathic engagement in females, where increased sensitivity to conspecific distress leads to hyper sociability without impairing the cognitive evaluation of novel social stimuli. In contrast, the absence of sociability deficits in vicarious males indicates a sex-dependent threshold for socially transmitted stress effects, consistent with reports that females display greater susceptibility to affective components of social stress. Importantly, the lack of significant impairment in social novelty in vicarious groups suggests that prefrontal circuit dysfunction does not globally disrupt social cognition but instead selectively impacts motivational and affective dimensions of social interaction. These findings support the idea that empathy-like processing may drive behavioral vulnerability in females, aligning with the observed female-specific alterations in *Oxtr* expression and synaptic scaffolding proteins.

Beyond helplessness, the VLH paradigm induced a broad syndromic depression. We observed significant anhedonia, evidenced by reduced sucrose preference and body weight changes. Female vicarious mice exhibited a significant reduction in sucrose preference at the endpoint, indicating that observational stress is sufficient to impair consummatory hedonic processing in females. Furthermore, both sexes exhibited increased anxiety-like behavior in the EPM, although this effect was statistically stronger in females than in vicarious males. The presence of behavioral despair in the FST across both sexes further validates the depressive-like phenotype induced by witnessing trauma.

Our interpretations align with, and extend, prior research on socially transmitted stress and depression-like phenotypes. Like Warren et al. (2013) we found increased CORT in observers, indicating that purely emotional trauma is sufficient to engage the HPA axis. Our analysis identified critical molecular targets shared by both sexes. First, we observed transcriptional upregulation of *Il6* in the PFC of both male and female vicarious mice. Elevated *Il6* is a reproducible biomarker of stress and depression in both humans and rodents, often linked to the activation of central immune signaling by psychological stress (Saxton et al., 2011; Ting et al., 2020). This suggests that the initial neuroinflammatory trigger is common to both sexes. Second, we identified the metabotropic glutamate receptor 2 (mGluR2) as a shared target of stress pathology at the protein level. We observed a significant downregulation of mGluR2 protein in the PFC of both male and female vicarious mice. mGluR2 serves as an autoreceptor that inhibits excessive glutamate release; its downregulation is associated with stress-induced excitotoxicity and impaired cognitive flexibility (Feyissa et al., 2010; Matosin et al., 2014). In male mice, the molecular footprint of vicarious stress aligned with the classical neurotrophic hypothesis of depression. We observed a significant downregulation of *Bdnf* mRNA and protein specifically in the male vicarious group. BDNF is essential for neuronal survival, synaptic plasticity, and dendritic maintenance (Matsuda et al., 2009; Vigers et al., 2012; Bathina and Das, 2015). Its reduction in the PFC is a well-characterized consequence of chronic stress in males, often leading to dendritic atrophy and synaptic loss.

The resilience of BDNF levels in females highlights a fundamental difference in how the female brain responds to psychological stress. Concurrently, males exhibited a specific downregulation of *Nr3c1* (Glucocorticoid Receptor) mRNA. The Glucocorticoid Receptor is critical for the negative feedback loop that terminates the HPA axis stress response. The loss of *Nr3c1* expression in males suggests a failure of this feedback mechanism, which likely perpetuates HPA hyperactivity and elevated corticosterone levels a mechanism supported by human genetic studies showing sex-specific associations between NR3C1 variants and HPA axis reactivity (Kumsta et al., 2007). In contrast to males, female mice maintained normal levels of *Bdnf* and *Nr3c1* but exhibited distinct deficits in social and structural proteins. Most notably, females showed a significant downregulation of the *Oxtr* mRNA. Oxytocin signaling in the PFC is critical for social buffering, anxiety regulation, and the modulation of fear responses (Li K. et al., 2016). The specific loss of *Oxtr* in females implies that vicarious stress compromises the social brain network, potentially removing the protective capacity of social bonding and rendering females more susceptible to despair and anxiety.

Females exhibited a specific loss of postsynaptic density proteins at the translational level. We observed significant downregulation of PSD-95 and SHANK3 proteins in the PFC of vicarious females. While males exhibited similar trends toward downregulation, these reductions did not reach statistical significance. These scaffolding proteins are crucial for stabilizing glutamate receptors and maintaining synaptic strength (Coley and Gao, 2019). Furthermore, we observed a unique downregulation of mature IL-1β protein in females, despite the concurrent upregulation of *Il6* mRNA. IL-1β plays a complex role in synaptic plasticity; while chronic elevation is neurotoxic, basal levels are required for memory formation and LTP. The suppression of mature IL-1β specifically in females could indicate a disruption in inflammasome processing or a compensatory mechanism attempting to limit excitotoxicity, but ultimately contributing to synaptic dysfunction. Importantly, several behavioral outcomes were conserved across sexes or varied depending on the behavioral test, whereas most of the molecular alterations showed sex-dependent divergence, suggesting that similar behavioral phenotypes may arise from partially distinct underlying biological mechanisms.

A key advantage of the VLH paradigm is its methodological uniformity across sexes, enabling direct delineation of sex-specific neurobehavioral mechanisms under identical experimental conditions. In conventional vicarious social defeat stress (VSDS) paradigms, aggression is typically driven by male CD1 residents; consequently, both male and female observer mice often witness male conspecifics being physically defeated, introducing cross-sex observational asymmetry and limiting true female-to-female stress modeling (Saxton et al., 2011; Ting et al., 2020). The VLH model overcomes this limitation by implementing an equivalent multisensory observational framework in which stress exposure is standardized within each sex, avoiding confounds arising from sex-biased aggression paradigms.

## 5 Limitations and future perspectives

This study validates the VLH paradigm as a potent model of vicarious stress induced depression, demonstrating that observing conspecific distress is sufficient to induce a robust depressive-like phenotype and HPA axis hyperactivity comparable to direct physical stress. Although group sizes were consistent with commonly used ranges in behavioral neuroscience, some assays included fewer than ten animals due to the constrained post-stress testing window prior to tissue collection, which may modestly limit the detection of subtle effects. Future circuit-level studies integrating ACC, insular, amygdalar, and hippocampal networks will be essential to delineate how empathy-related processing interfaces with executive dysfunction to drive vicarious depression-like phenotypes. Given the sex-dependent molecular signatures observed, spatial transcriptomics could further identify laminar or subregion-specific adaptations within the PFC. Ultimately, these data are suggestive of the urgent need to move beyond “male-biased” neurobiology, advocating for sex-stratified therapeutic strategies that target the specific molecular vulnerabilities driving depression in males and females.

## Data Availability

The original contributions presented in this study are included in this article/Supplementary material, further inquiries can be directed to the corresponding author.
