# Supplementary material for: Vicarious learned helplessness: a translationally relevant novel model of stress contagion elucidating sex-dependent prefrontal cortex pathology
Source: Front Behav Neurosci. 2026 Mar 5;20:1788847. doi: 10.3389/fnbeh.2026.1788847 (PMC12999966; doi:10.3389/fnbeh.2026.1788847)
Supplement: Supplementary file 1 [file Data_Sheet_1.pdf]

SUPPLEMENTARY

Vicarious learned helplessness: a translationally relevant novel model of stress contagion elucidating sex-dependent prefrontal cortex pathology

Shashikant Patel[1,3], Roli Kushwaha[1,3], Debiprasad Sinha[1,3], Kalyani Soren[1,3], Arvind Kumar[1,2], Sumana Chakravarty[1,3]*

[1]Department of Applied Biology, CSIR-Indian Institute of Chemical Technology, Hyderabad, India
[2]CSIR-Centre for Cellular and Molecular Biology (CSIR-CCMB), Hyderabad, India
[3]Academy of Scientific and Innovative Research (AcSIR), Ghaziabad 201002, Uttar Pradesh, India

*Corresponding Author:
Dr. Sumana Chakravarty
Applied Biology, CSIR- Indian Institute of Chemical Technology (IICT),
Tarnaka, Hyderabad- 500 007 (A.P.)
Email: sumanachak@csiriict.in; sumana98@gmail.com,
Phone: +91 040 27193155
FAX: 040 27193189
ORCID ID: https://orcid.org/0000-0001-8223-6508

Sup_Table 1. Primer sequences used for quantitative real-time PCR

Gene Symbol	Gene Name	Forward Primer (5′→3′)	Reverse Primer (5′→3′)
Nr3c1	Glucocorticoid Receptor	ACCTGGAAGCTCGAAAAACGA	CAGCAGTGACACCAGGGTAG
Il6	Interleukin-6	TACCACTTCACAAGTCGGAGGC	CTGCAAGTGCATCATCGTTGTTC
Oxtr	Oxytocin Receptor	TGGCGGTCCTGTGTCTCATACTG	CGACATCAGCAACAGCAGGTAGG
Esr2	Estrogen Receptor-β	TCTGCCAAGGAGACTCGCTACT	GGTGCATTGGTTTGTAGCTGGAC
Casp3	Caspase-3	TGTCATCTCGCTCTGGTACG	TCCCATAAATGACCCCTTCA
Bdnf	Brain-Derived Neurotrophic Factor	CCGAGCTCATCTTTGCCAGA	GAAGCAGCTTTCTCAACGCC
Gapdh	Glyceraldehyde-3-phosphate dehydrogenase	GGAGAGTGTTTCCTCGTCCC	ATGAAGGGGTCGTTGATGGC

Sup_Table 2. Primary and secondary antibodies used for immunoblotting

S.No	Name	Catalog No.	Dilution
1	TNF-alpha	AMC3012, Invitrogen	1:1000
2	BDNF	Ab108319, abcam	1:1000
3	IL-1B	P420B, Invitrogen	1:1000
4	PSD95	MA1-046, Invitrogen	1:1000
5	α-Tubulin	Ab15246, abcam	1:1000
6	mGLUR2	D7D8M, CST	1:1000
7	SHANK3	D5K6R, CST	1:1000
8	NMDAR2A	Ab133265, abcam	1:1000
7	Anti-rabbit Secondary	G21234, Invitrogen	1:5000
8	Anti mouse Secondary	G21040, Invitrogen	1:5000

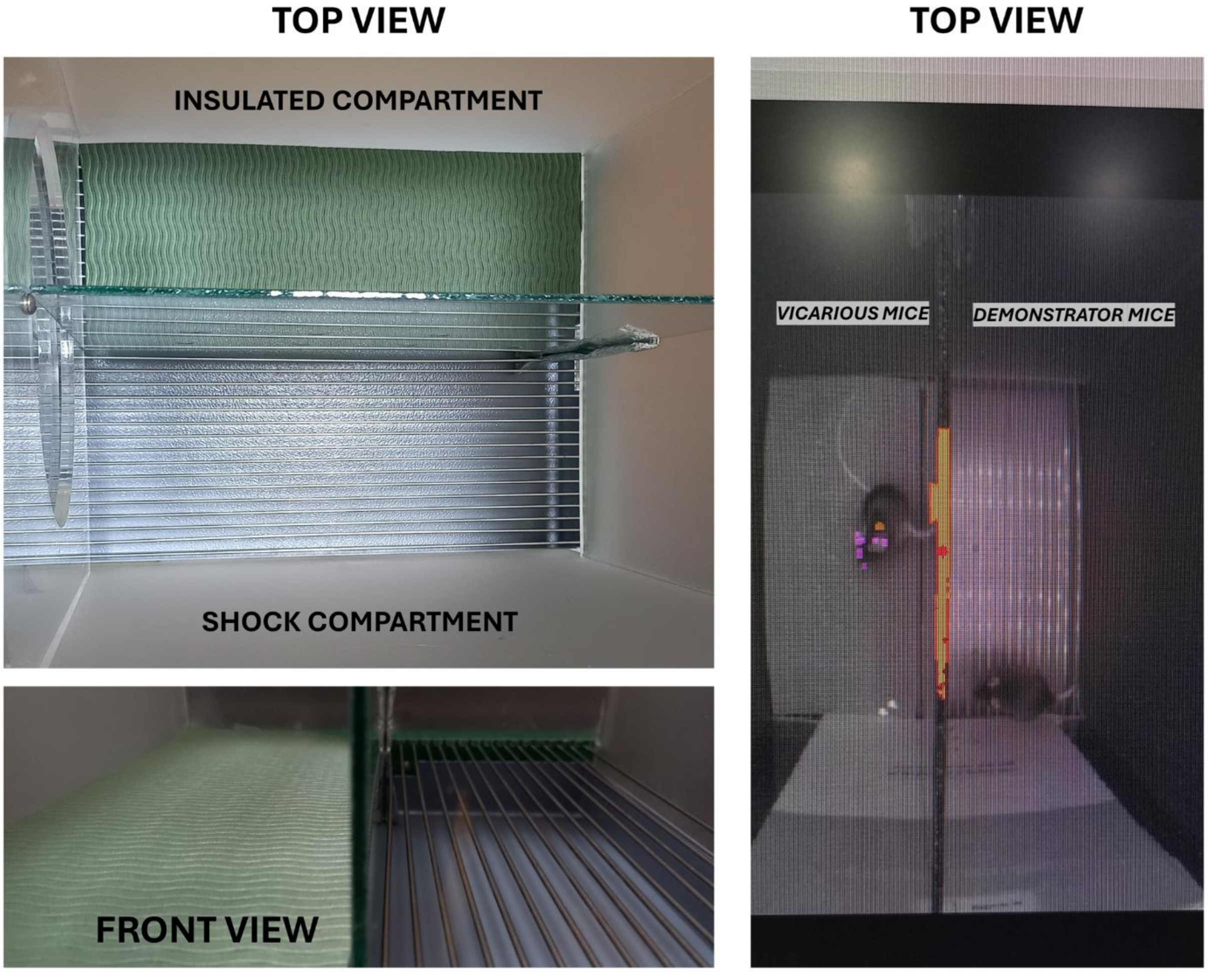

Sup_Figure 1: Representative images of the modified fear-conditioning chamber used for the VLH paradigm. The apparatus consists of two equal compartments separated by a transparent, perforated glass partition that permits visual, auditory, and olfactory interaction while preventing physical contact. One compartment contains a stainless-steel grid floor connected to a programmable shock generator for delivery of inescapable foot shocks to demonstrator mice, whereas the adjacent compartment is electrically insulated to house vicarious mice. This configuration enables purely observational stress exposure. The same apparatus was subsequently adapted for the modified active avoidance test by allowing controlled access between compartments, enabling quantification of escape latency and avoidance behavior.

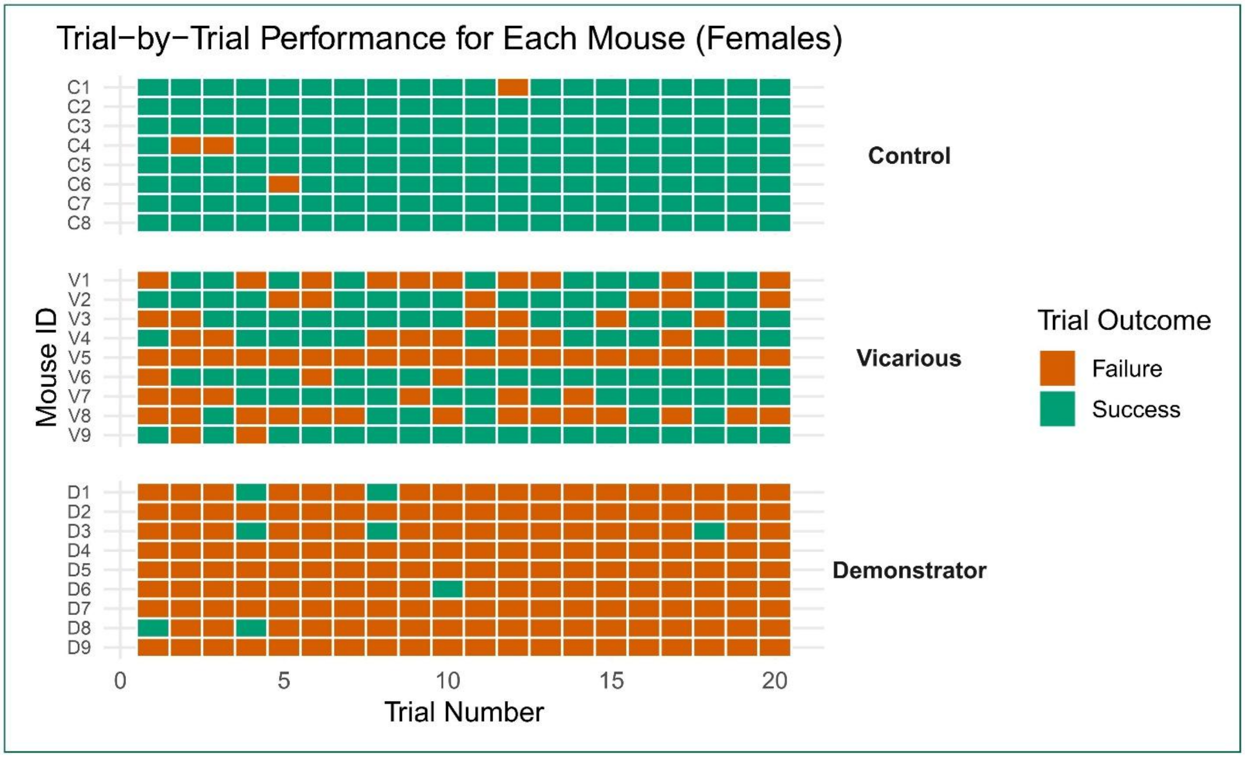

Sup_Figure 2: Individual Trial-by-Trial Escape Performance in Female Mice
Detailed behavioral trajectories of female mice in the modified active avoidance test. Individual mice failure/ success for control (n=8), vicarious (n=9), and demonstrator (n=8) groups is plotted across 20 consecutive trials.

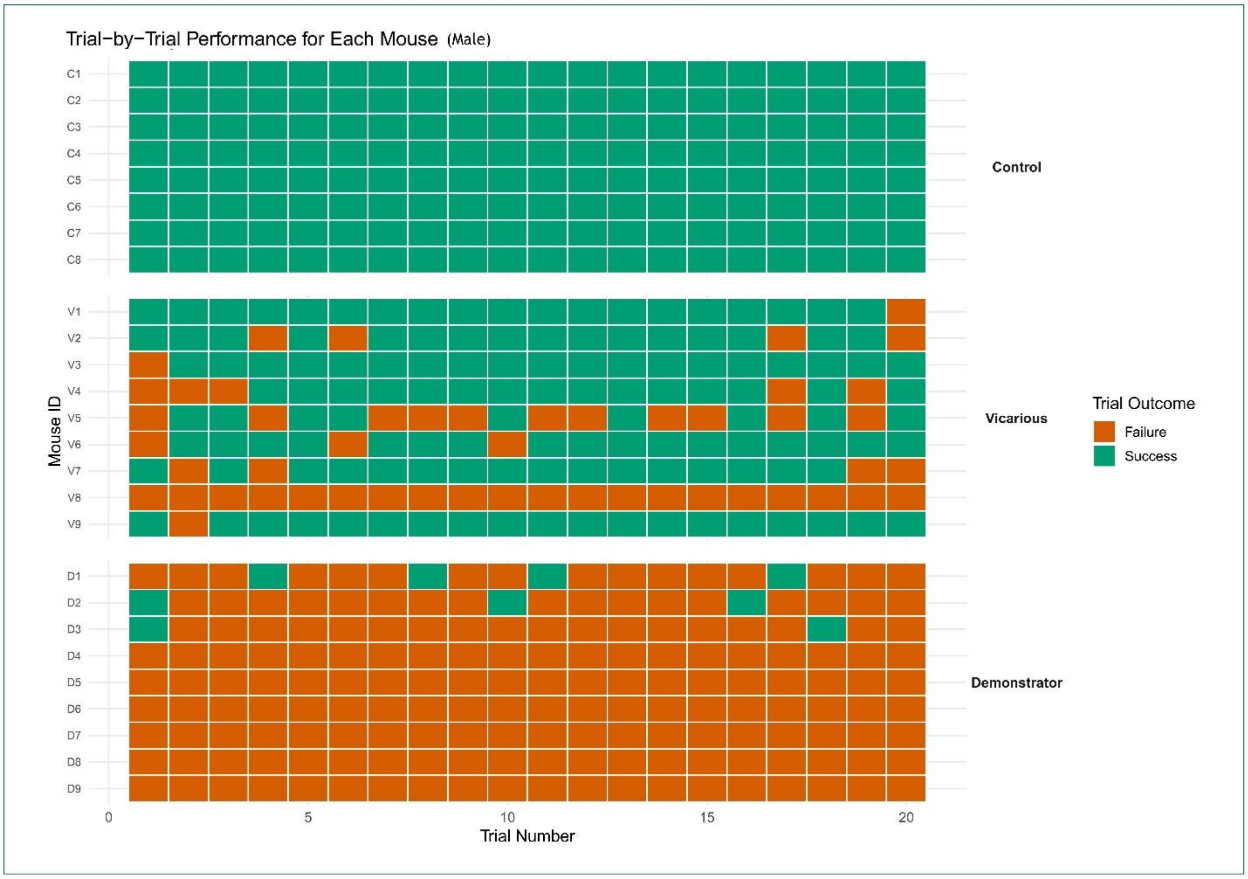

Sup_Fig 3: Individual Trial-by-Trial Escape Performance in Male Mice

Detailed behavioral trajectories of male mice in the modified active avoidance test. individual performance for control (n=8), vicarious (n=9), and demonstrator (n=9) groups is plotted across 20 consecutive trials.

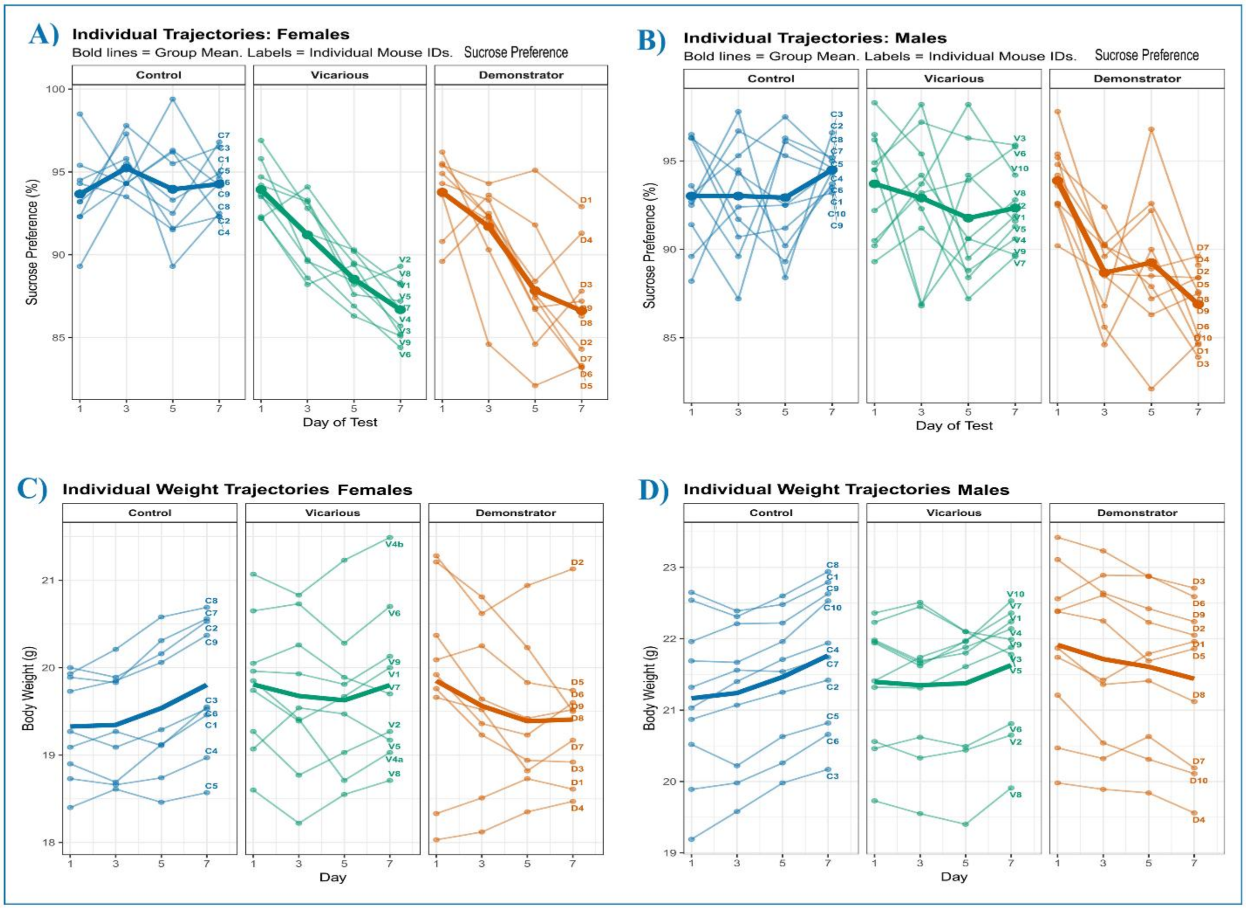

Sup_Fig 4: Individual Trajectories of Sucrose Preference and weight changes over Time
Longitudinal tracking of anhedonia-like behavior across the 7-day experimental paradigm. Lines represent the individual sucrose preference (%) trajectories for each mouse. (A) Females: control mice maintain high, stable preference, while both vicarious and demonstrator females show progressive, significant declines from Day 1 to Day 7. (B) Males: Stability is maintained in control and vicarious groups, whereas demonstrator males show a selective and significant reduction in preference over time. (C) and (D) represent individual mice weight change trajectory over 7 days of female and male mice respectively.

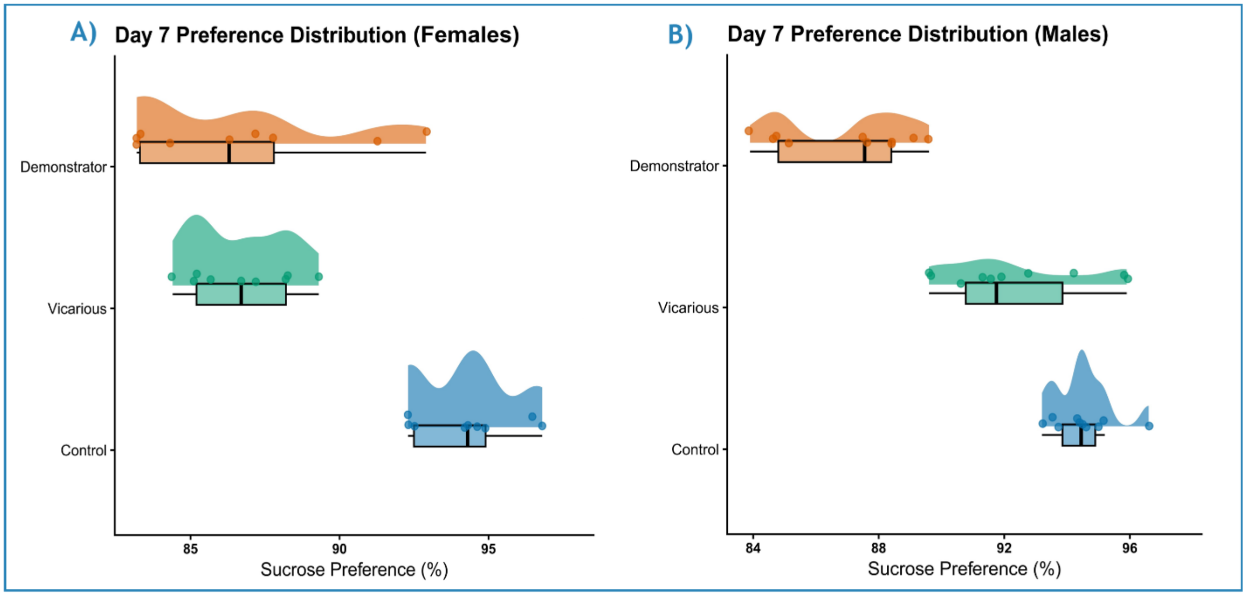

Sup_Fig 5: Boxplots represent the median and interquartile range (IQR) of sucrose preference (%) for each group on Days 1, 3, 5, and 7 for females and males.

SUPPLEMENTARY RESULTS:

1. Probability analysis in task acquisition reliability following stress

To assess the consistency of task acquisition, we analyzed the probability of escape across the 20 trials (Fig 3C). This metric represents the percentage of animals in each group that successfully avoided the shock (latency < 60 s) at each specific trial. A probability approaching 100% indicates that the behavior has been reliably learned and adopted by the entire cohort, whereas a probability near 0% reflects a persistent failure to respond, indicative of behavioral inhibition or learned helplessness. Female control mice initiated the session with a probability of 100% (Trial 1), experienced a minor dip during the exploratory phase (Trial 2: 87.5%), and re-established perfect reliability by the final block (Trial 16–20: 100%). Conversely, female demonstrator mice showed negligible success, starting with a low probability of 12.5% at Trial 1 and rapidly dropping to 0% by Trial 2, an effect that persisted through Trial 20. The female vicarious group (based on overall success rates) exhibited an intermediate but poor profile (approx. 56% average probability), indicating that social stress exposure prevents the formation of a reliable, consistent avoidance strategy. Male control mice demonstrated perfect reliability; the probability of escape was 100% from the very first trial and remained at this ceiling throughout the entire session. In contrast, the male demonstrator group exhibited a near-zero probability of escape (e.g., Trial 1: ~11%; Trial 20: 0%), confirming that the vast majority of these animals never engaged in the avoidance behavior. The Male vicarious group displayed an unstable behavioral profile, with escape probabilities fluctuating significantly across the session (averaging ~73% overall), suggesting that while some learning occurred, the strategy was not reliably consolidated across the group.

2. Efficiency of Successful Avoidance Responses

To assess the efficiency of the avoidance behavior when it was successfully executed, we analyzed the mean latency exclusively for trials where animals successfully escaped (latency < 60 s) (Fig. 3D). This metric distinguishes between animals that escape slowly versus those that fail to escape entirely. The results indicated that even when socially stressed animals managed to escape, they did so less efficiently than controls. Male control mice exhibited the fastest reaction times, with a mean latency of 23.8 s (± 2.14 SEM) on successful trials. In contrast, male vicarious mice were significantly slower, averaging 40.4 s (± 2.51 SEM) per successful escape. Male demonstrator mice had so few successful escapes that their data point relies on sparse data, averaging 47.1 s (± 1.91 SEM). A similar trend was observed in females. Female control mice averaged 26.5 s (± 2.45 SEM) per successful escape. Female vicarious mice were notably slower, with a mean latency of 42.0 s (± 2.66 SEM), and the female demonstrator group (based on rare successful trials) averaged 45.0 s (± 2.78 SEM). These findings suggest that social stress exposure impairs not only the probability of responding but also the cognitive processing speed or motor initiation required for efficient avoidance.
